# Efficient synthesis of piperazinyl amides of 18β-glycyrrhetinic acid

**DOI:** 10.3762/bjoc.16.73

**Published:** 2020-04-21

**Authors:** Dong Cai, ZhiHua Zhang, Yufan Meng, KaiLi Zhu, LiYi Chen, ChangXiang Yu, ChangWei Yu, ZiYi Fu, DianShen Yang, YiXia Gong

**Affiliations:** 1 College of Public Basic Sciences, Jinzhou Medical University, Jinzhou, 121001, China; 2 School of Chemical and Environmental Engineering, Liaoning University of Technology, Jinzhou, 121001, China; 3 College of Pharmacy, Jinzhou Medical University, Jinzhou, 121001, China; 4 College of Pharmacy, Jiamusi University, Jiamusi, 154007, China

**Keywords:** 18β-glycyrrhetinic acid, piperazinyl amides, synthesis

## Abstract

In the present study, a practical method to prepare piperazinyl amides of 18β-glycyrrhetinic acid was developed. Two main procedures for the construction of important intermediate **8** are discussed. One procedure involves the amidation of 1-Boc-piperazine with 3-acetyl-18β-glycyrrhetinic acid, prepared by the reaction of 18β-glycyrrhetinic acid with acetic anhydride without any solvent at 130 °C. The other procedure to prepare compound **8** involves the amidation of 18β-glycyrrhetinic acid followed by the esterification with acetic anhydride. Finally, compound **8** underwent *N*-Boc deprotection to prepare product **4**. To ascertain the scope of the reaction, another C-3 ester derivative **17** was tested under the optimized reaction conditions. Furthermore, the reasons for the appearance of byproducts were elucidated. Crystallographic data of a selected piperazinyl amide is reported.

## Introduction

**Figure 1:**
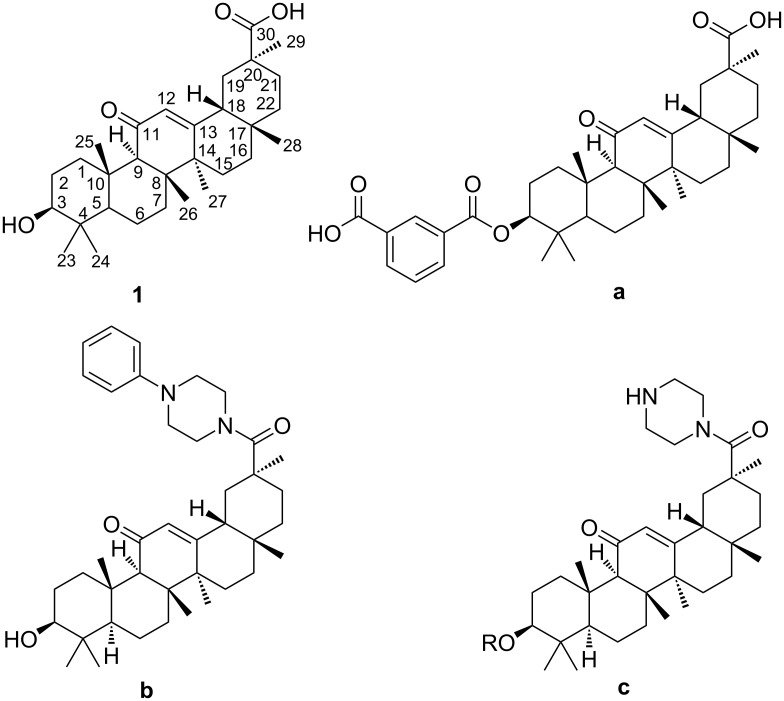
Chemical structure of 18β-glycyrrhetinic acid and known derivatives.

Glycyrrhizin was the major bioactive component in Glycyrrhiza uralensis fisch root. 18β-glycyrrhetinic acid (**1**, Figure 1) was then obtained by hydrolysis of glycyrrhizin. 18β-glycyrrhetinic acid and its derivatives have been extensively investigated in medicinal chemistry for their various biological activities, including anti-inflammatory [1], antiulcer [2], antioxidative [3], antitumor [4], and antibacterial [5] activity and their proberties as hepatic protective agents [6].

Over the past few decades, the emphasis was placed primarily on explaining structure–activity relationships of 18β-glycyrrhetinic acid derivatives to enhance their biological activity. The structure of 18β-glycyrrhetinic acid offered numerous possibilities to chemical modifications, thereby leading to the formation of novel derivatives [7–9]. There are three functional groups at C-3, C-11 and C-30 in the structure of 18β-glycyrrhetinic acid amenable to chemical modifications.

The modifications at the C3-OH group of 18β-glycyrrhetinic acid are identified to be relatively common and effective. The modification of the C3-OH group, altering the molecular polarity of 18β-glycyrrhetinic acid, may be an advantage in achieving better cytotoxicity or antiproliferative activity [8–10]. For instance, the hydroxy group can be converted into an oxime, acyloxyimino, alkoxyimino, alkoxy and 3-oxo group [9]. As a proteasome inhibitor, compound **a** suppresses the chymotrypsin-like activity of the proteasome in MT4 cells with an IC_50_ of 0.22 μM, nearly 100-fold more potent than 18β-glycyrrhetinic acid.

Besides, the C-30 carboxyl group is often esterified or amidated in order to enhance the antitumor or other efficacy of 18β-glycyrrhetinic acid derivatives [11]. A novel piperazinyl amide **b** exhibits the optimal inhibitory activity against MCF-7 and can be further developed as a potent VEGFR2 and antitumor agent [12].

Piperazinyl amide fragments have the ability to form several hydrogen bonds, modulate the acid–base equilibrium constant and change the octanol–water partition coefficient [13]. They are considered as the basic motif for designing many biologically active molecules [14–15]. Some piperazinyl amides of 18β-glycyrrhetinic acid (**c**) are synthesized using various methods. A method involves the C30-position of the acyl chloride with symmetric piperazine [16–17]. In this case, the acyl chloride can be prepared without purification, and the total yield over two steps can reach 81% [16]. Such structures (**c**) can also be formed by treating the C30 carboxyl group with piperazine in the presence of activators (e.g., 1-ethyl-3-(dimethylaminopropyl)carbodiimide hydrochloride (EDCl), 1-hydroxybenzotriazole (HOBt) and trimethylamine [18]), the yield of piperazinyl amide derivatives was 80.6% [19]. These two methods are complicated through the side formation of bisamide, obviously, due to the competitive attack on the N atoms of the symmetric diamine. An alternative method [12] to prepare such piperazinyl amides (**c**) involves the amidation of 18β-glycyrrhetinic acid with 4-substituted phenylpiperazines in the presence of *N,N*-dicyclohexylcarbodiimide (DDC) and HOBt. Under such reaction conditions, the bisamide as byproduct can be evitable, whereas the substituted piperazine compounds will not be readily available.

In principle, an efficient approach to the synthesis of structural analogues of (**c**) is based on the reaction of carboxylic acid with *N*-Boc-protected aliphatic diamine in the presence of activators and then deprotection of the *tert*‐butoxycarbonyl (Boc) group using trifluoroacetic acid (TFA) [20]. Compared with the mentioned methods, the latter method achieves the higher yield (up to 90%) without the formation of byproducts [21].

Given the flexible 18β-glycyrrhetinic acid scaffold in the design of biologically active compounds, a novel and efficient method was reported to prepare C3 ester derivatives or/and C30 piperazinyl amides (**c**).

18β-Glycyrrhetinic acid belongs to the class of ursane-type pentacyclic triterpenoids, and the synthetic methods studied here can also apply to the modification of structurally similar other triterpenoic acids, but further experimental verification is needed.

## Results and Discussion

**Scheme 1:**
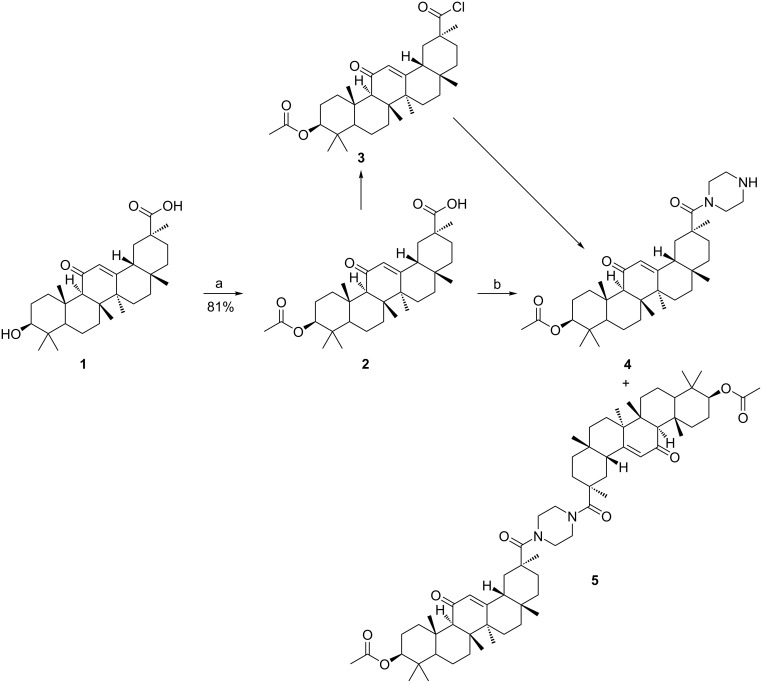
Synthesis of compound **4**. Reagents and conditions: (a) Ac_2_O, NEt_3_, DMF (cat.), DCM, 25 °C, 1 day; (b) oxalyl chloride, NEt_3_, DMF (cat.), DCM, 25 °C, 5 h, then piperazine, DCM, NEt_3_, DMAP, 0/25 °C, 30 min.

The synthetic route to 18β-glycyrrhetinic acid piperazinyl amide **4** was originally reported by Sommerwerk [16] (Scheme 1). In this case, 18β-glycyrrhetinic acid reacted with acetic anhydride in the presence of triethylamine to give 3-acetyl-18β-glycyrrhetinic acid (**2**), which by successive chlorination with oxalyl chloride yielded acyl chloride **3**. Without isolation, the intermediate **3** reacted with piperazine to give 18β-glycyrrhetinic acid piperazinyl amide **4**. The total yield of chlorination and amidation reactions was 67%.

In the beginning, the reaction of acyl chloride **3** with piperazine was taken as a prototypical case to ascertain and screen the experimental conditions. First, the solution with intermediate **3** was cooled to 0 °C, and triethylamine (1.1 equiv) as well as piperazine (3.0 equiv) were added. After 30 min of stirring, the solvent was removed in vacuo and the residue was purified by column chromatography to afford amide **4** and bisamide **5**. When increasing the amount of piperazine, relatively high yields of amide **4** would be achieved (Table 1, entries 1–3). Given the high reactivity of the acyl chloride with piperazine, we decided to verify the feasibility of the feeding sequence in a model system. The solution with intermediate **3** was added dropwise to a solution of piperazine in 30 mL of dichloromethane (DCM). After the solutions were mixed, intermediate **3** was surrounded by piperazine molecules. The excess of piperazine could sufficiently avoid the formation of bisamide **5** (Table 1, entry 4). When intermediate **3** was dissolved in more DCM (100 mL), less side reactions occurred and the product was obtained in higher yield (Table 1, entries 4 and 5). By altering the amidation reaction temperature, the yield of amide **4** showed an obvious variation. A reaction temperature led to excessive bisamide **5** (Table 1, entry 6). The results of the experiments demonstrated that the side reaction was inevitable, and the amount of bisamide **5** produced was dependent on the reaction temperature and material ratio. The isolation and purification of product **4** were significantly more difficult, causing an unavoidable loss of target compound as well.

**Table 1:** Formation of piperazinyl amide **4**: selected optimization reactions.

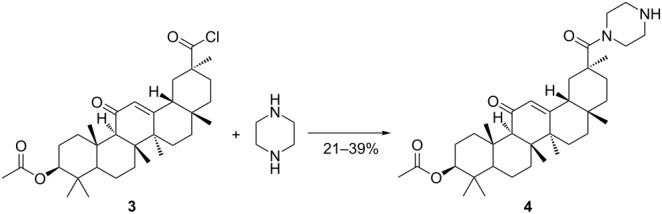		

entry	conditions^a^	yield of **4**^b^

1	piperazine (3 equiv), 0 °C	21%
2	piperazine (6 equiv), 0 °C	28%
3	piperazine (10 equiv), 0 °C	33%
4	compound **3** (in 30 mL DCM) was added dropwise to the solution of piperazine (10 equiv), 0 °C	36%
5	compound **3** (in 100 mL DCM) was added dropwise to the solution of piperazine (10 equiv), 0 °C	39%
6	compound **3** (in 100 mL DCM) was added dropwise to the solution of piperazine (10 equiv), 25 °C	32%

^a^Reaction performed on a 0.90 mmol scale of acyl chloride **3**. ^b^Isolated yield.

**Scheme 2:**
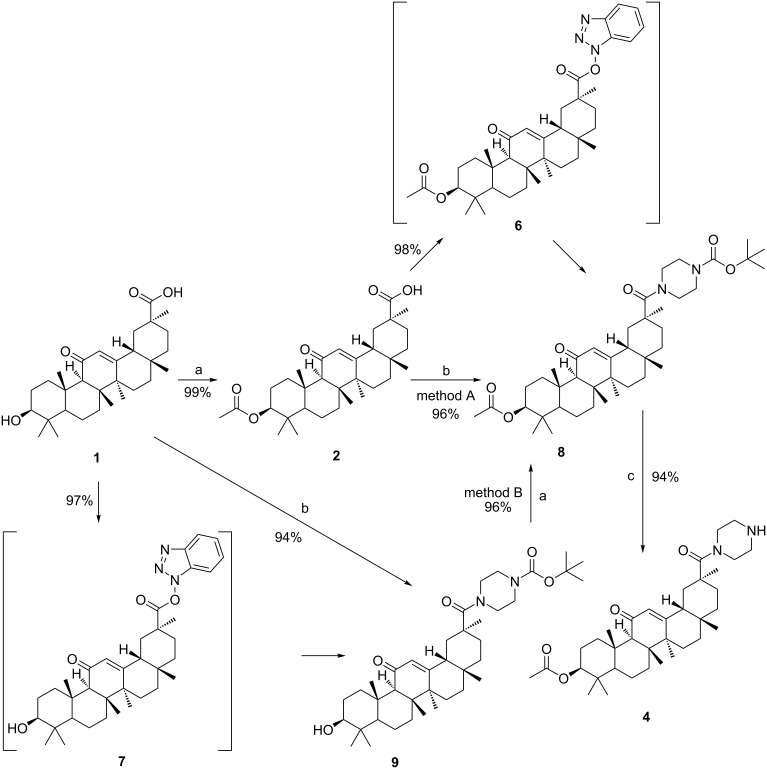
Synthesis of compound **4**. Reagents and conditions: (a) Ac_2_O, 130 °C, 1 h; (b) 1-Boc-piperazine, CH_3_CN, NEt_3_, EDCl, HOBt, reflux, 24 h; (c) TFA, DCM, 0/25 °C.

The observation described in Scheme 1 and Table 1 led us to consider a new approach for preparing the piperazinyl amide **4** (Scheme 2). Two general procedures for the synthesis of compound **8** are documented. One method involves acetylation of 18β-glycyrrhetinic acid, followed by amidation of the resulting ester **2** to give compound **8**. Finally, the piperazinyl amide **4** was synthesized by of N-Boc deprotection.

Another procedure for the synthesis of compound **8** involves acylation of compound **9**, which was prepared by the reactions of 18β-glycyrrhetinic acid with 1-Boc-piperazine under similar reaction conditions. Compound **8** was prepared in virtually quantitative yield from compound **9** with acetic anhydride without solvent at 130 °C. The acetic acid formed in the reaction need not be neutralized and can escape from the reaction system. The reaction gave the optimal yield at 125–135 °C. When the temperature was below 125 °C, compound **9** did not reacted completely after 48 h (TLC monitoring).

The reaction from **2** to **8** proceeded via the intermediate **6**, which can be isolated. The intermediate **6** did not react with 1-Boc-piperazine in low boiling point solvents even after an extended reaction time (up to 72 h). Heating the reaction using conventional or microwave methods had no effect on the conversion of intermediate **6** to compound **8**.

**Figure 2:**
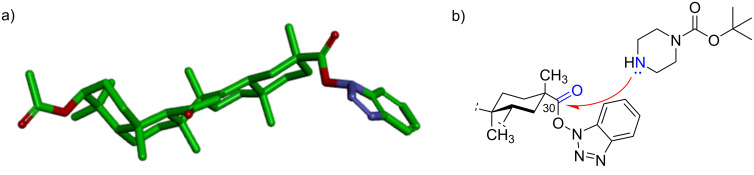
a) Estimated structure of the intermediate **6**; b) Possible aminolysis process.

To explain the stable structure of the intermediate **6** (Figure 2a), energy minimization by MM2 was performed using the ChemBio3D Ultra 14.0 (CambridgeSoft Corporation, 2014) software force field [22]. The estimated structure was supported by crystal structures of similar structural compounds [23]. Figure 2b suggests that the steric hindrance around the C30 ester group of intermediate **6** has a remarkable influence on the aminolysis reaction. The mechanism of the aminolysis reaction revealed that a higher reaction temperature of the solvent mixture contributed to the synthesis of compound **8**.

To promote the yield of compound **8** and improve the overall yield of the reaction, an optimization of the reaction conditions was performed (Table 2). First, by using low boiling point solvents, only low or no yields (<19%) of the desired compound **8** were obtained after a longer reaction time (Table 2, entries 1–4). A further investigation of the reaction using microwave irradiation to heat at reflux temperature also failed to produce the target product (Table 2, entry 2). The intermediate **6** exhibited very low solubility in ethyl acetate solution, thereby directly causing the incomplete conversion of the substrate over several days (Table 2, entry 5). The reaction performed in acetonitrile as solvent achieved an excellent yield within 10 h but still with a small amount of intermediate **6** (Table 2, entry 6). Besides, *N,N*-dimethylformamide (DMF), a solvent with a higher boiling point than acetonitrile, was also tested, yet the reaction led to the formation of unknown compounds (TLC monitoring, Table 2, entry 7).

**Table 2:** Optimization of the reaction conditions for the coupling of compound **2** with 1-Boc-piperazine.

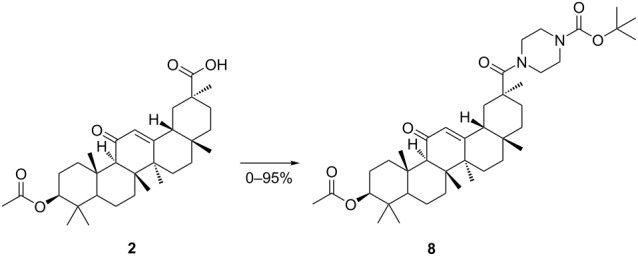				

entry	solvent	conditions^a^	yield of **6**^b^	yield of **8**^b^

1	CH_2_Cl_2_	reflux, 48 h	93%	trace
2	CH_2_Cl_2_	reflux, 48 h, microwave	93%	trace
3	acetone	reflux, 48 h	93%	trace
4	THF	reflux, 48 h	81%	19%
5	EtOAc	reflux, 48 h	37%	61%
6	acetonitrile	reflux, 10 h	12%	88%
7	DMF	reflux, 5 h, 130 ^o^C	0	0
8	acetonitrile	reflux, 10 h, 1-Boc-piperazine (2.5 mmol)	0	95%

^a^**2** (1.0 mmol), EDCl (1.2 mmol), NEt_3_ (1.2 mmol), HOBt (1.2 mmol) and 1-Boc-piperazine (1.2 mmol) were dissolved in 10 mL of solvent for the indicated time. ^b^Isolated yield.

After screening several solvents, the optimal yield was obtained in acetonitrile, though a small amount of unconverted intermediate **6** was observed in the reaction mixture (Table 2, entry 6). Next, the reactivity of compound **2** with 1-Boc-piperazine in a 1:1.5 ratio was investigated. The reaction did not proceed completely in acetonitrile at reflux temperature, even after the period was prolonged to 48 h. In contrast, 1-Boc-piperazine (2.5 equiv) exhibited high performance, thereby leading to the preparation of compound **8** in 95% yield (Table 2, entry 8), which essentially completed conversion of the intermediate **6** within 10 h.

Alternatively, the aminolysis reaction of the isolated intermediate **6** with 1-Boc-piperazine (2.5 equiv) without any other additives can readily proceed in acetonitrile at reflux temperature in 95% yield.

**Scheme 3:**
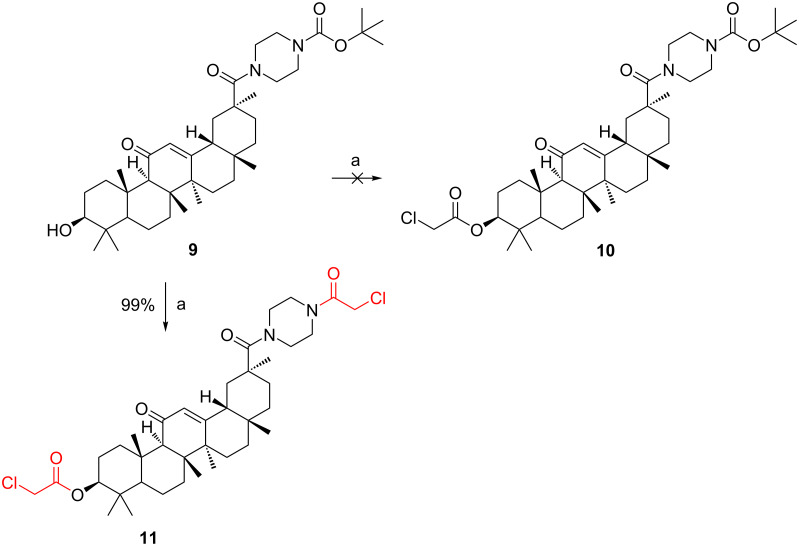
Synthesis of byproduct 11. Reagents and conditions: (a) chloroacetic anhydride, 130 °C, 1 h.

Having demonstrated the effectiveness of the new procedure, we decided to apply this method towards the synthesis of compound **10**. But our investigations proved that the above experimental conditions seem not to be suitable for the synthesis of compound **10**. Interestingly, treatment of compound **9** with chloroacetic anhydride by heating did not afforded compound **10**, but obtained another compound, **11** (Scheme 3).

In our analysis, compound **9** reacted with acetic anhydride to give compound **8** and acetic acid. At the reaction temperature, the acetic acid (boiling point 117–118 °C) boiled and vapors escaped from the reaction mixture during the reaction process. However, in the reaction of compound **9** with chloroacetic anhydride (Scheme 3), the chloroacetic acid (boiling point 189 °C) formed during the reaction process cannot escape from the reaction system in the form of a gas, therefore, the initially formed compound **10** undergo *N*-Boc deprotection in an acidic medium to give byproduct **11**. The structure of the byproduct **11** was confirmed by HRMS analysis.

Another noteworthy factor is that the acidity of chloroacetic acid (p*K*_a_ = 2.87) was stronger than that of acetic acid (p*K*_a_ = 4.76). The procedure for the generation of byproduct **11** was similar to *N*-Boc-deprotection using trifluoroacetic acid.

The effective synthesis of compound **10** was then explored (Table 3). In our initial attempt, compound **9** was reacted with chloroacetic anhydride (Table 3, entry 1) at 130 °C, whereas the reaction was complicated by the side formation of byproduct **11**, which was obviously due to the presence of chloroacetic acid. Next, the reactions were performed with organic or inorganic bases to tie up or neutralize the chloroacetic acid released in the reaction. The base was provided in an amount or in a slight excess corresponding to the quantity required to tie up or neutralize the theoretical amount of acid released. The excess base did not affect the reaction and was washed off with water after the reaction. Triethylamine is a weak organic base that cannot completely neutralize the acid generated in the reaction. It may also volatilize at this reaction temperature (Table 3, entry 2). When the inorganic base was used as an acid-binding agent, the H_2_O, produced during the neutralization reaction, consumed considerable quantities of chloroacetic anhydride (Table 3, entries 3 and 4). Thus, when the amount of chloroacetic anhydride was elevated to 20 equiv, compound **9** were not completely converted within 48 h under reflux conditions. Next, compound **9** was reacted with chloroacetic anhydride in refluxing toluene in the presence of K_2_CO_3_ in a Dean–Stark apparatus to remove the water formed during the reaction (Table 3, entry 5). Under such conditions, the reaction was completed in 1 h to give product **15**, though a small amount of byproduct **11** was observed in the reaction mixture. Examples of suitable inorganic bases include Na_2_CO_3_, NaHCO_3_, and especially K_2_CO_3_. The synthesis of compound **9** was described in a similar manner in [11].

**Table 3:** Formation of amide **10**: selected optimization reactions.

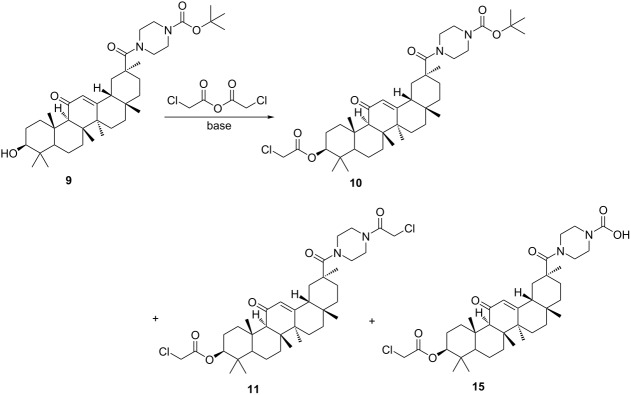				

entry	conditions^a^	yield of **10**^b^	yield of **11**^b^	yield of **15**^b^

1	chloroacetic anhydride (10 equiv)	0	99	0
2	chloroacetic anhydride (10 equiv), NEt_3_, acetonitrile	0	90	trace
3	chloroacetic anhydride (10 equiv), K_2_CO_3_, acetonitrile	0	34	trace
4	chloroacetic anhydride (20 equiv), K_2_CO_3_, acetonitrile	0	36	trace
5	chloroacetic anhydride (8 equiv), K_2_CO_3_, toluene, water separator	0	trace	96
6	chloroacetic anhydride (8 equiv), Na_2_CO_3_, toluene, water separator	0	trace	92
7	chloroacetic anhydride (8 equiv), NaHCO_3_, toluene, water separator	0	trace	91

^a^Reaction performed on a 10 mmol scale. ^b^Isolated yield.

In another reaction, 18β-glycyrrhetinic acid was smoothly esterified with chloroacetic anhydride at the C3-OH group to form compound **12** in a quantitative yield. The reaction was completed in 30 min. The principal advantages of this method are that there is no solvent required, and that the reaction time is shorter than in previous cases [16], since the acid formed during the reaction may catalyze the esterification reaction of 18β-glycyrrhetinic acid with chloroacetic anhydride.

**Scheme 4:**
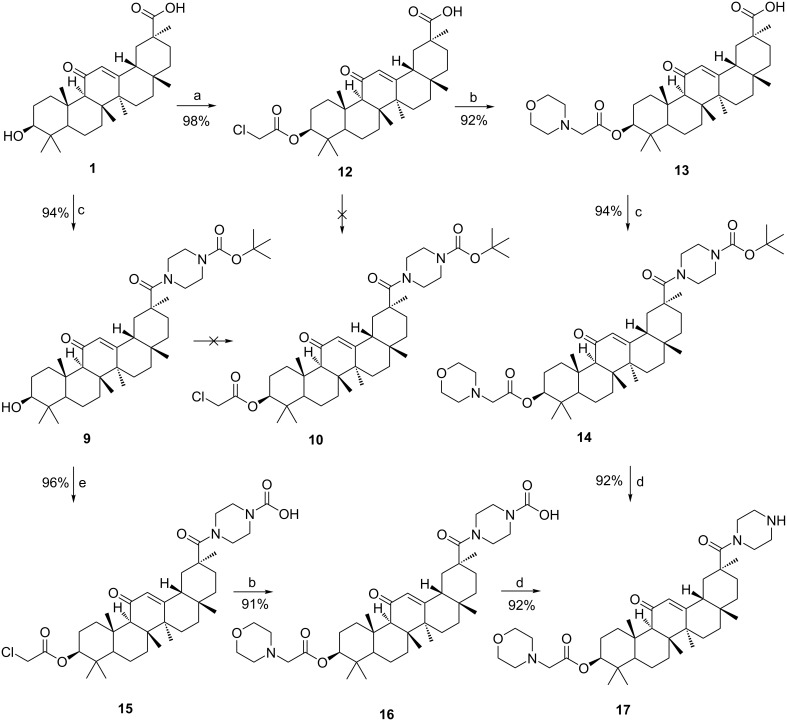
Synthesis of compound **17**. Reagents and conditions: (a) chloroacetic anhydride, 130 °C, 1 h; (b) morpholine, K_2_CO_3_/ I_2_, C_2_H_5_OH; (c)1-Boc-piperazine, CH_3_CN, NEt_3_, EDCl, HOBt, reflux, 24 h; (d) TFA, DCM, 0/25 °C; (e) chloroacetic anhydride, K_2_CO_3_, toluene, 130 °C, 1 h.

Having accessed compound **12**, many other new compounds can also be prepared by replacing an amine with the halogen atom. With compounds **12** and **15** in hand, we then tried to synthesize target compound **17** (Scheme 4), which is a novel 18β-glycyrrhetinic acid derivative to biologically active substances.

According to the first method, compound **12** reacted with morpholine in the presence of K_2_CO_3_/I_2_ to give compound **13**, followed by successive amidation and *N*-Boc deprotection gave compound **17**. It is noteworthy that the carboxyl group of compound **12** did not react with inorganic bases, such as K_2_CO_3_, Na_2_CO_3_, NaHCO_3_, etc.

Another approach for the synthesis of compound **17** is the amination of compound **15** to give compound **16**. Subsequently, compound **17** was obtained by hydrolysis of compound **16** using TFA in DCM. The synthesis of compounds **15**–**17** was described in a similar manner in [11].

**Figure 3:**
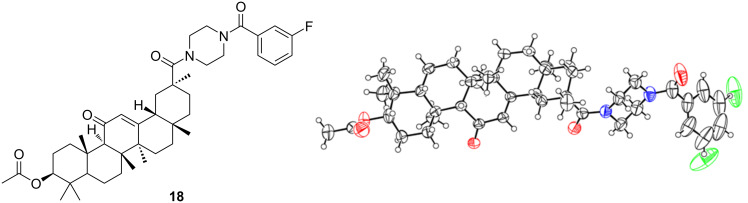
Crystal structure of conpound **18**.

After the C3 position has been converted to a stable ester structure, the piperazinyl amides of 18β-glycyrrhetinic acid can be also converted to novel unsymmetrical amides. For example, treatment of compound **4** with acyl chloride in dry DCM under alkaline conditions directly afforded 18β-glycyrrhetinic acid derivative **18**. The molecular structure of compound **18** in the crystalline state is shown in Figure 3. In this crystal structure, there is an orientational disorder of the *m*-fluorophenyl moiety due to the rotation of a single bond. Details for the crystal structure determinations are given in Table 4.

**Table 4:** Crystallographic data of compound **18**.

CCDC number	1904891
formula	C_43_H_59_FN_2_O_5_
formula weight	702.92
crystal colour, shape	colourless
Solution	CH_3_OH
crystal system	monoclinic
space group	P2_1_2_1_2_1_
*a* / Å	11.581 (3)
*b* / Å	12.216 (3)
*c* / Å	27.749 (7)
α (°)	90
β (°)	90
γ (°)	90
*V* / Å^3^	3925.7 (17)
*T* / K	273
*Z*	4
density (calculated) / g·cm^−3^	1.189
*F*(000)	1520
*R* (reflections)	0.0439 (5028)
*wR^2^* (reflections)	0.1130 (7118)

## Conclusion

To sum up, piperazinyl amides of 18β-glycyrrhetinic acid were prepared from the 18β-glycyrrhetinic acid or its corresponding acyl chloride and piperazine. In this case, a considerable number of byproducts were inevitably produced. To reduce and/or avoid the formation of byproducts, efficient procedures were developed to prepare 18β-glycyrrhetinic acid derivatives that contain a terminal piperazinyl amide fragment. Due to the steric hindrance around the C30 ester group of intermediates, the intermediates **6** and **8** do not react with 1-Boc-piperazine at lower reaction temperature. The results showed the temperature dependence of the reaction between 18β-glycyrrhetinic acid and 1-Boc-piperazine. Besides, the Boc-piperazinyl amides of 18β-glycyrrhetinic acid reacted with chloroacetic anhydride in the presence of a base to give product **15**. If no base is present, the Boc-piperazinyl amide of 18β-glycyrrhetinic acid **9** will undergo *N*-Boc deprotection and amidation to give compound **11**. These results can be used to produce *N*-substituted piperazines of 18β-glycyrrhetinic acid in high yield, and further to develop more asymmetric piperazinamide derivatives. The structure of the asymmetric piperazinyl amide was confirmed by X-ray analysis.

## Experimental

Detailed synthetic procedures for all new compounds including copies of their NMR spectra can be found in Supporting Information File 1.

## Supporting Information

File 1 Preparation procedures and analytical data of compounds **1**, **4**–**9**, **11**, **13**, **15**–**18**.
