# Supplementary material for: Efficient synthesis of piperazinyl amides of 18β-glycyrrhetinic acid
Source: Beilstein J Org Chem. 2020 Apr 21;16:798–808. doi: 10.3762/bjoc.16.73 (PMC7188925; doi:10.3762/bjoc.16.73)
Supplement: File 1 — Preparation procedures and analytical data of compounds 1, 4–9, 11, 13, 15–18. [file Beilstein_J_Org_Chem-16-798-s001.pdf]

BEILSTEIN JOURNAL OF ORGANIC CHEMISTRY

# Supporting Information

for

# Efficient synthesis of piperazinyl amides of 18β-glycyrrhetinic acid

Dong Cai, ZhiHua Zhang, Yufan Meng, KaiLi Zhu, LiYi Chen, ChangXiang Yu, ChangWei Yu, ZiYi Fu, DianShen Yang and YiXia Gong

*Beilstein J. Org. Chem.* **2020,** *16,* 798–808. doi:10.3762/bjoc.16.73

# Preparation procedures and analytical data of compounds 1, 4–9, 11, 13, 15–18

License and Terms: This is a supporting information file under the terms of the Creative Commons Attribution License (http://creativecommons.org/licenses/by/4.0). Please note that the reuse, redistribution and reproduction in particular requires that the authors and source are credited.
The license is subject to the *Beilstein Journal of Organic Chemistry* terms and conditions: (https://www.beilstein-journals.org/bjoc)

**Table of contents**

Synthetic procedures……………...…...……………...……………..……………...………....…S1–17
$^{1}H$ and $^{13}C$ NMR and HRMS of compound (**1**) ……...………...…...……...………...………...…S19
$^{1}H$ and $^{13}C$ NMR and HRMS of compound (**8**) ……...………...……..………………...………...…S20
$^{1}H$ and $^{13}C$ NMR and HRMS of compound (**12**) ……...………...……...……………...……......S22
$^{1}H$ and $^{13}C$ NMR and HRMS of compound (**6**) ……...………...………...……………………...…S23
$^{1}H$ and $^{13}C$ NMR and HRMS of compound (**7**) ……...………...………...………….....…….…S25
$^{1}H$ and $^{13}C$ NMR and HRMS of compound (**9**) ……...………...………...……...…...…….…S26
$^{1}H$ and $^{13}C$ NMR and HRMS of compound (**14**) ……...………...………...……………...…….…S28
$^{1}H$ and $^{13}C$ NMR and HRMS of compound (**11**) ……………………...……...……………...…S29
$^{1}H$ and $^{13}C$ NMR and HRMS of compound (**15**) ……...………...………...………...……....…S31
$^{1}H$ and $^{13}C$ NMR and HRMS of compound (**13**) ……...………...………...…...………...…...…S32
$^{1}H$ and $^{13}C$ NMR and HRMS of compound (**16**) ……...………...………...…...………...…...…S34
$^{1}H$ and $^{13}C$ NMR and HRMS of compound (**4**) ……...………...………...…………….………S35
HRMS of compound (**5**) ……...………...………...………………...………...………...………...…S36
$^{1}H$ and $^{13}C$ NMR and HRMS of compound (**17**) ……...………...………...…...………...………S37
$^{1}H$ and $^{13}C$ NMR and HRMS of compound (**18**) ……...………...………...…...………...………S38

# Experimental

## Materials and methods

Unless otherwise required, all reagents used in the experiment were purchased as commercial analytical grade and used without further purification. Melting points were obtained in open capillary tubes with a WRS-1B melting point apparatus and were uncorrected (Shen Guang Electric Appliances Co., Ltd., Shanghai, CHN). The structures of the synthetic compounds were confirmed by $^{1}$H NMR and $^{13}$C NMR spectra on 400/54Premium Shielded NMR Magnet System (Agilent Technologies, Santa Clara, CA, USA) with tetramethylsilane (TMS) as an internal standard. HRMS spectral data were collected from an Agilent 6200 Series TOF and 6500 Series Q-TOF LC/MS System B.05.01. (B5125) and Thermo Scientific LTQ-Orbitrap XL in positive ion modes (Agilent Technologies, Santa Clara, CA, USA). X-ray single-crystal structure determinations were carried out on a Bruker SMART APEX II CCD diffractometer (Bruker AXS GMBH, Karlsruhe, GER).

## Synthesis of 18β–GA analogs

*3β-Acetoxy-11-oxo-18β-olean-12-en-30-oic acid* (**2**)

18β-Glycyrrhetinic acid (0.47 g, 1.0 mmol) was heated at 130 $^{o}$C with acetic anhydride (2.04 g, 20 mmol) for 1 h. Then, $H_2O$ was added to the cool solution. The product was filtered off and washed with cold $H_2O$.

A white solid; yield, 99.2%; m.p. 304.4.0-306.1 °C (literature [1]: 312.0-313.0 °C); $^1$H NMR (400 MHz, Chloroform-*d*) δ 5.69 (d, *J* = 1.7 Hz, 1H, CH-12), 4.50 (dt, *J* = 11.7, 2.8 Hz, 1H, CH-3), 2.77 (dd, *J* = 14.0, 4.0 Hz, 1H, CH-1), 2.35 (s, 1H, CH-9), 2.03 (s, 2H, acetyloxy $CH_3$), 1.35 (m, 3H, $CH_3$-27), 1.21 (s, 3H, $CH_3$-25), 1.14 (s, 3H, $CH_3$-26), 1.10 (s, 3H, $CH_3$-29), 0.86 (s, 6H, $CH_3$-23/24), 0.85 (s, 3H, $CH_3$-28), 0.77 (m, 1H, CH-5); $^{13}$C NMR (101 MHz, Chloroform-*d*) δ 200.41 (C11), 181.77 (30), 171.08 (acetyloxy C=O), 169.55 (C13), 128.37 (C12), 80.61 (C3), 61.66 (C9), 54.97 (C5), 48.21 (C18), 45.44 (C14), 43.79 (C20), 43.17 (C8), 40.78 (C19), 38.73 (C1), 38.02 (C4), 37.67 (C22), 36.89 (C10), 32.66 (C7), 31.84 (C17), 30.86 (C21), 28.52 (C29), 28.44 (C28), 28.02 (C23), 26.43 (C2), 26.35 (C15), 23.54 (C16), 23.32 (C27), 21.32 (acetyloxy $CH_3$), 18.64 (C26), 17.33 (C6), 16.67 (C25), 16.39 (C24). HRMS (*m*/*z*): [M + H]$^+$ calcd. for $C_{32}H_{49}O_5$: 513.3580, found: 513.3580.

General procedure for the preparation of compounds (**4**) and (**5**)

The compound **3** (0.44 g, 0.90 mmol) was dissolved in $CH_2Cl_2$ (30 mL) at 0 °C under stirring, then triethylamine (0.3 g, 3.00 mmol) and anhydrous piperazine (0.23 g, 2.70 mmol) were added. The reaction was stirred at 0 °C for 30 min. After reaction, the mixture was removed, and the residue was subjected to column chromatography (silica gel, $CH_2Cl_2$-methanol, 5:1) to yield compounds (**4**) and (**5**).

*3β-Acetoxy-11-oxo-18β-olean-12-en-30-carbonyl piperazine* (**4**) A white solid; yield, 36.1%; m.p. 237.2-239.0 °C (literature [2]: 160 °C-decomp.); $^1$H NMR (400

MHz, Chloroform-*d*) δ 5.67 (s, 1H, CH-12), 4.49 (dd, *J* = 11.7, 4.8 Hz, 1H, CH-3), 3.61 (q, *J* = 4.7 Hz, 4H, piperazine $CH_2$×2), 2.85 (t, *J* = 5.0 Hz, 4H, piperazine $CH_2$×2), 2.77 (dt, *J* = 13.7, 3.6 Hz, 1H, CH-1), 2.33 (s, 1H, CH-9), 2.29 – 2.23 (m, 1H, CH-16), 2.16 (s, 3H, acetyloxy $CH_3$), 1.33 (s, 3H, $CH_3$-27), 1.20 (s, 3H, $CH_3$-25), 1.13 (s, 3H, $CH_3$-26), 1.10 (s, 3H, $CH_3$-29), 0.85 (s, 6H, $CH_3$-23/24), 0.79 (s, 3H, $CH_3$-28), 0.76 (m, 1H, CH-5); $^{13}$C NMR (101 MHz, Chloroform-*d*) δ 200.01 (C11), 173.86 (C30), 171.03 (acetyloxy C=O), 169.67 (C13), 128.46 (C12), 80.59 (C3), 61.67 (C9), 54.99 (C5), 48.21(C18), 46.13 (piperazine C×2), 45.27 (C14), 43.77 (C20), 43.71 (C8), 43.26 (piperazine C×2), 38.78 (C19), 38.01 (C1/4), 37.73 (C22), 36.91 (C10), 33.38 (C7), 32.72 (C17), 31.76 (C21), 28.41 (C29), 28.02 (C28), 27.02 (C23), 26.69 (C2), 26.41 (C15), 23.54 (C16), 23.07 (C27), 21.32 (acetyloxy $CH_3$), 18.65 (C26), 17.35 (C6), 16.66 (C25), 16.41 (C24); HRMS (*m*/*z*): [M + H]$^+$ calcd. for $C_{36}H_{57}N_2O_4$: 581.4318, found: 581.4316.

*Bisamide* (**5**) A white solid; yield, 64.6%; m.p. 211.4-212.0 °C. HRMS (*m*/*z*): [M + Na]$^+$ calcd. for $C_{68}H_{102}N_2NaO_8$: 1097.7534, found: 1097.7535.

*3β-Acetyloxy -11-oxo-18β-olean-12-en-30-carbonyl piperazine* (**4**)

Compound **8** (0.68 g, 1.0 mmol) was dissolved in $CH_2Cl_2$ (10 mL) at 0 °C under stirring. Trifluoroacetic acid (5 mL) was added, and the reaction was stirred at 0 °C for 3 h. After reaction, the mixture was made basic with a saturated $Na_2CO_3$ solution. This mixture was extracted with $CH_2Cl_2$, dried over $Na_2SO_4$, and concentrated to give the desired product.

A white solid; yield, 94.1%; the chemical structures were characterized as above.

*1H-Benzo[d][1,2,3]triazol-1-yl-3β-acetoxy-11-oxo-olean-12-en-30-oate* (**6**)

Compound **2** (0.51 g, 1.0 mmol) was dissolved in acetonitrile (20 mL), then EDCl (0.23 g, 1.2 mmol), triethylamine (0.13 g, 1.2 mmol) and HOBt (0.16 g, 1.2 mmol) were added. The mixture was stirred at room temperature for 1 h. The solvent was removed under vacuum to give a residue which was treated with a mixture of ethanol and water. The solution was stirred at room temperature for 30 min, and a solid was obtained by filtration while washing with $H_2O$.

A white solid; yield, 97.8%; m.p. 208.7 °C - decomp. (literature [3]: 192-195 °C, decomp.); $^1$H NMR (400 MHz, Chloroform-*d*) δ 8.08 (d, *J* = 8.4 Hz, 1H, phenyl), 7.56 (t, *J* = 7.6 Hz, 1H, phenyl), 7.44 (t, *J* = 7.7 Hz, 1H, phenyl), 7.34 (d, *J* = 8.3 Hz, 1H, phenyl), 5.71 (s, 1H, CH-12), 3.22 (dd, *J* = 10.7, 5.5 Hz, 1H, OH-3), 2.77 (dt, *J* = 13.5, 3.6 Hz, 1H, CH-1), 2.39 – 2.23 (m, 2H, CH-9/16), 1.41 (s, 3H, $CH_3$-27), 1.15 (s, 3H, $CH_3$-25), 1.13 (s, 3H, $CH_3$-26), 1.00 (s, 3H, $CH_3$-29), 0.93 (s, 3H, $CH_3$-23), 0.80 (s, 3H, $CH_3$-24), 0.72 (d, *J* = 11.6 Hz, 1H, CH-5); $^{13}$C NMR (101 MHz, Chloroform-*d*) δ 199.91 (C11), 172.51 (C30), 167.60 (C13), 143.54 (phenyl), 129.02 (phenyl), 128.83 (phenyl), 128.54 (C12), 124.82 (phenyl), 120.66, (phenyl) 107.81 (phenyl), 78.70 (C3), 61.83 (C9), 54.89 (C5), 48.20 (C18), 45.36 (C20), 44.36(C8), 43.15(C19), 40.85 (C1), 39.11 (C4), 37.75 (C22), 37.06 (C10), 32.72 (C7), 31.97 (C17),

31.16 (C21), 28.54 (C29), 28.08 (C28), 28.02 (C23), 27.25 (C2), 26.34 (C15/16), 23.48 (C27), 18.67 (C26), 17.45 (C6), 16.34 (C25), 15.57 (C24); HRMS (*m*/*z*): $[M + H]^+$ calcd. for $C_{36}H_{50}N_3O_4$: 588.3801, found: 588.3801.

*1H-Benzo[d][1,2,3]triazol-1-yl-3β-hydroxy-11-oxo-olean-12-en-30-oate* (**7**)

18β-Glycyrrhetinic acid (0.47 g, 1.0 mmol) was dissolved in acetonitrile (20 mL), then EDCl (0.23 g, 1.2 mmol), triethylamine (0.13 g, 1.2 mmol) and HOBt (0.16 g, 1.2 mmol) were added. The mixture was stirred at room temperature for 1 h. The solvent was removed under vacuum to give a residue which was treated with a mixture of ethanol and water. The solution was stirred at room temperature for 30 min, and a solid was obtained by filtration while washing with $H_2O$.

A white solid; yield, 97.2%; m.p. 263.4-264.4 °C; $^1$H NMR (400 MHz, Chloroform-*d*) δ 8.09 (d, *J* = 8.4 Hz, 1H, phenyl), 7.57 (t, *J* = 7.6 Hz, 1H, phenyl), 7.44 (t, *J* = 7.7 Hz, 1H, phenyl), 7.35 (d, *J* = 8.3 Hz, 1H, phenyl), 5.72 (s, 1H, CH-12), 4.52 (dd, *J* = 11.6, 4.8 Hz, 1H, CH-3), 2.78 (dt, *J* = 13.8, 3.7 Hz, 1H, CH-1), 2.37 (s, 1H, CH-9), 2.05 (s, 3H, acetyloxy $CH_3$), 1.41 (s, 3H, $CH_3$-27), 1.21 (s, 3H, $CH_3$-25), 1.16 (s, 6H, $CH_3$-26/29), 0.94 (s, 3H, $CH_3$-23), 0.88 (s, 6H, $CH_3$-24/28), 0.80 (m, 1H, CH-5); $^{13}$C NMR (101 MHz, Chloroform-*d*) δ 199.76 (C11), 172.51 (30), 171.01 (acetyloxy C=O), 167.60 (C13), 143.55 (phenyl), 128.99 (phenyl), 128.81 (phenyl), 128.54 (C12), 124.80 (phenyl), 120.67 (phenyl), 107.80 (phenyl), 80.53 (C3), 61.74 (C9), 54.98 (C5), 48.19 (C18), 45.37 (C14), 44.35 (C20), 43.14 (C8), 40.84 (C19), 38.74 (C1), 38.02

(C4), 37.74 (C22), 36.91 (C10), 32.67 (C7), 31.97 (C17), 31.17 (C21), 28.53 (C29), 28.02 (C28/23), 26.33 (C2/15), 23.52 (C16), 23.42 (C27), 21.32 (acetyloxy $CH_3$), 18.67 (C26), 17.34 (C6), 16.66 (C25), 16.38 (C24); HRMS (*m*/*z*): (M + $H^+$) calcd. for $C_{38}H_{52}N_3O_5$: 630.3907, found: 630.3904.

*tert-Butyl 4-(3β-acetoxy-11-oxo-18β-olean-12-en-30-carbonyl)piperazine-1-carboxylate* (**8**)

Method A: Compound **2** (0.51 g, 1.0 mmol) was dissolved in acetonitrile (20 mL), then EDCI (0.23 g, 1.2 mmol), triethylamine (0.13 g, 1.2 mmol) and HOBt (0.16 g, 1.2 mmol) were added.The mixture was stirred at room temperature for 20 min. The 1-Boc-piperazine (0.46 g, 2.5 mmol) was added, and the mixture was stirred under reflux for 10 h. The solvent was removed under vacuum to give a residue which was treated with a mixture of ethanol and water. The solution was stirred at room temperature for 30 min, and a solid was obtained by filtration while washing with $H_2O$.

Method B: Compound **9** (0.64 g, 1.0 mmol) was heated at 130 °C with acetic anhydride (2.04 g, 20 mmol) for 1 h. Then, $H_2O$ was added to the cool solution. The product was filtered off and washed with cold $H_2O$.

A white solid; yield, 95.7%; m.p. 221.6-223.0 °C; $^1$H NMR (400 MHz, Chloroform-*d*) δ 5.66 (s, 1H, CH-12), 4.50 (dd, *J* = 11.7, 4.7 Hz, 1H, CH-3), 3.66 – 3.50 (m, 4H, piperazine $CH_2$×2), 3.40 (d, *J* = 5.1 Hz, 4H, piperazine $CH_2$×2), 2.79-2.74 (m, 1H, CH-1), 2.33 (s, 1H, CH-9), 2.30 – 2.22 (m, 1H, CH-16), 2.03 (s, 3H, acetyloxy $CH_3$), 1.45 (s, 9H, tert-butyl $CH_3$×3), 1.33 (s, 3H, $CH_3$-27), 1.20 (s, 3H,

$CH_3$-25), 1.14 (s, 3H, $CH_3$-26), 1.09 (s, 3H, $CH_3$-29), 0.85 (s, 6H, $CH_3$-23/24), 0.79 (s, 3H, $CH_3$-28), 0.76 (m, 1H, CH-5); $^{13}$C NMR (101 MHz, Chloroform-*d*) δ 199.95 (C11), 174.12 (C30), 171.03 (acetyloxy C=O), 169.46 (C13), 154.52 (Boc C=O), 128.50 (C12), 80.58 (tert-butyl C), 80.24 (C3), 61.67 (C9), 54.99 (C5), 48.12 (C18), 45.27 (C14), 43.87 (piperazine C×2), 43.75 (C20), 43.25 ( C8/19), 38.77 (C1/C4), 38.01 (piperazine C×2), 37.70 (C22), 36.90 (C10), 33.22 (C7), 32.72 (C17), 31.75 (C21), 28.40 (C29), 28.35 (tert-butyl $CH_3$×3), 28.02 (C28), 27.05 (C23), 26.68 (C2), 26.38 (C15), 23.54 (C16), 23.08 (C27), 21.31 (acetyloxy $CH_3$), 18.65 (C26), 17.35 (C6), 16.66 (C25), 16.40 (C24); HRMS (*m*/*z*): $[M + H]^+$ calcd. for $C_{41}H_{65}N_2O_6$: 681.4843, found: 681.4841.

*tert-Butyl 4-(3β-hydroxyl-11-oxo-18β-olean-12-en-30-carbonyl)piperazine-1-carboxylate* (**9**)

18*β*-Glycyrrhetinic acid (0.47 g, 1.0 mmol) was dissolved in acetonitrile (20 mL), then EDCI (0.23 g, 1.2 mmol), triethylamine (0.13 g, 1.2 mmol) and HOBt (0.16 g, 1.2 mmol) were added. The mixture was stirred at room temperature for 20 min. The 1-Boc-piperazine (0.46 g, 2.5 mmol) was added, and the mixture was stirred under reflux for 10 h. The solvent was removed under vacuum to give a residue which was treated with a mixture of ethanol and water. The solution was stirred at room temperature for 30 min, and a solid was obtained by filtration while washing with $H_2O$.

A white solid; yield, 94.3%; m.p. 224.3-225.7 °C; $^1$H NMR (400 MHz, Chloroform-*d*) δ 5.66 (s, 1H, CH-12), 3.63-3.52 (m, 4H, piperazine $CH_2$×2), 3.39 (t, *J* = 5.2

Hz, 4H, piperazine $CH_2$×2), 3.22-3.18 (m, 1H, OH-3), 2.79-2.74 (m, 1H, CH-1), 2.31 (s, 1H, CH-9), 2.30-2.23 (m, 1H, CH-16), 1.45 (s, 9H, tert-butyl $CH_3$×3 ), 1.34 (s, 3H, $CH_3$-27), 1.20 (s, 3H, $CH_3$-25), 1.11 (s, 3H, $CH_3$-26), 1.10 (s, 3H, $CH_3$-29), 0.98 (s, 3H, $CH_3$-23), 0.79 (s, 3H, $CH_3$-24), 0.78 (s, 3H, $CH_3$-28), 0.68 (d, *J* = 11.6 Hz, 1H, CH-5); $^{13}$C NMR (101 MHz, Chloroform-*d*) δ 200.10 (C11), 174.13 (C30), 169.40 (C13), 154.53 (Boc C=O), 128.56 (C12), 80.25 (C3), 78.75 (tert-butyl C), 61.77 (C9), 54.92 (C5), 48.08 (C18), 45.26 (C14), 43.88 (C20), 43.82 (piperazine C×2), 43.26 (C8/C19), 39.12 (C1/C4), 39.10 (piperazine C×2), 37.70 (C22), 37.06 (C10), 33.16 (C7), 32.79 (C17), 31.75 (C21), 28.40 (C29), 28.36 (tert-butyl $CH_3$×3), 28.07 (C28), 27.28 (C23), 27.05 (C2), 26.69 (C15), 26.39 (C16), 23.14 (C27), 18.66 (C26), 17.46 (C6), 16.36 (C25), 15.56 (C24); HRMS (*m*/*z*): $[M + H]^+$ calcd. For $C_{39}H_{63}N_2O_5$: 639.4737, found: 639.4736.

*N-(2-chloroacetyl) piperazinyl 3β-(2-chloroacetoxy)-11-oxo-18β-olean-12-en-29-amide* (**11**)

Compound **9** (0.64 g, 1.0 mmol) was heated at 130 °C with chloroacetic anhydride (3.42 g, 20 mmol) for 1 h. Then, $H_2O$ was added to the cool solution. The product was filtered, washed with cold $H_2O$ and dried.

A white solid; yield, 99%; m.p. 177.4-175.7 °C; $^1$H NMR (400 MHz, Chloroform-*d*) δ 5.67 (s, 1H, CH-12), 4.60 (dd, *J* = 11.8, 4.7 Hz, 1H, CH-3), 4.14 – 3.99 (m, 4H, piperazine $CH_2$×2), 3.73 – 3.66 (m, 4H, piperazine $CH_2$×2), 3.66 – 3.55 (m, 2H, $CH_2$-Cl), 3.53 (d, *J* = 5.4 Hz, 2H, $CH_2$-Cl), 2.81 (dt, *J* = 13.8, 3.7 Hz, 1H, CH-1), 2.35 (s, 1H, CH-9), 2.30 (dd, *J* = 13.4, 3.8 Hz,

1H, CH-16), 1.35 (m, 3H, $CH_3$-27), 1.23 (s, 3H, $CH_3$-25), 1.15 (s, 3H, $CH_3$-26), 1.11 (s, 3H, $CH_3$-29), 0.89 (s, 6H, $CH_3$-23/24), 0.81 (s, 3H, $CH_3$-28), 0.79 (m, 1H, CH-5); $^{13}$C NMR (101 MHz, chloroform-*d*) δ 199.81 (C11), 174.25 (C30), 169.44 (C13), 167.12 (chloroacetoxy C=O), 165.32 (chloroacetoxy C=O), 128.50 (C12), 82.97 (C3), 61.60 (C9), 54.93 (C5), 48.04 (C18), 46.27 (C14), 45.25 (piperazine C), 43.92 (piperazine C), 43.83 (C20), 43.26 (C8), 42.20 (C19), 41.24 (C1), 40.67 (C-Cl), 38.66 (C4), 38.21(piperazine C×2), 37.67 (C22), 36.86 (C10), 32.98 (C7), 32.66 (C17), 31.75 (C21), 28.39 (C29), 28.00 (C28), 27.02 (C23), 26.63 (C2), 26.34 (C15), 23.39 (C16), 23.10 (C27), 22.63, 18.64 (C26), 17.30 (C6), 16.60 (C25), 16.40 (C24); HRMS (*m*/*z*): $[M + H]^+$ calcd. for $C_{38}H_{57}Cl_2N_2O_5$: 691.3645, found: 691.3641.

*3β-(2-Chloroacetoxy)-11-oxo-18β-olean-12-en-30-oic acid* (**12**)

18β-Glycyrrhetinic acid (0.47 g, 1.0 mmol) was heated at 130 °C with chloroacetic anhydride (3.42 g, 20 mmol) for 1 h. Then, $H_2O$ was added to the cool solution. The product was filtered off and washed with cold $H_2O$.

A white solid; yield, 98.0%; m.p. 259.0 °C - decomp. (literature [1]: 260.8–261.8 °C); $^1$H NMR (400 MHz, Chloroform-*d*) δ 5.70 (s, 1H, CH-12), 4.59 (dd, *J* = 11.8, 4.8 Hz, 1H, CH-3), 4.05 (d, *J* = 2.3 Hz, 2H, $CH_2$-Cl), 2.81 (m, 1H, CH-1), 2.35 (s, 1H, CH-9), 2.17 (dd, *J* = 13.6, 4.1 Hz, 1H, CH-16), 1.36 (m, 3H, $CH_3$-27), 1.21 (s, 3H, $CH_3$-25), 1.15 (s, 3H, $CH_3$-26), 1.11 (s, 3H, $CH_3$-29), 0.88 (s, 6H, $CH_3$-23/24), 0.82 (s, 3H, $CH_3$-28) , 0.78 (m, 1H, CH-5); $^{13}$C NMR (101 MHz, Chloroform-*d*) δ 200.26 (C11), 181.51(C30), 169.59 (C13), 167.12 (acetyloxy

C=O), 128.37 (C12), 83.00 (C3), 61.60 (C9), 54.94 (C5), 48.21 (C18), 45.43 (C14), 43.78 (C20), 43.18 (C8), 41.24 (C19), 40.80 (C-Cl), 38.64 (C1), 38.23 (C4), 37.67 (C22), 36.87 (C10), 32.62 (C7), 31.84 (C17), 30.87 (C21), 28.52 (C29), 28.43 (C28), 28.00 (C23), 26.43 (C2), 26.34 (C15), 23.41 (C16), 23.35 (C27), 18.64 (C26), 17.30 (C6), 16.61 (C25), 16.39 (C24); HRMS (*m*/*z*): $[M + H]^+$ calcd. for $C_{32}H_{48}ClO_5$: 547.3190, found: 547.3188.

3*β-(2-Morpholinoacetoxy)-11-oxo-olean-12-ene-30-oic acid* (**13**)

Compound **12** (0.55 g, 1.0 mmol), morpholine (0.13 g, 1.5 mmol), $K_2CO_3$ (0.69 g, 5.0 mmol) and a catalytic amount of $I_2$ in absolute ethanol (15 mL) was stirred under reflux for 12 h. The reaction mixture was evaporated under reduced pressure and the residue was dissolved in ethanol/$H_2O$ mixture and the white precipitate was collected by filtration.

A white solid; yield, 92.0%; m.p.275.3-276.4 °C; $^1$H NMR (400 MHz, Chloroform-*d*) δ 5.72 (t, *J* = 3.0 Hz, 1H, CH-12), 4.60 (dd, *J* = 11.2, 5.5 Hz, 1H, CH-3), 3.76 (d, *J* = 4.9 Hz, 4H, morpholine), 3.26 – 3.20 (m, 2H, $CH_2$), 2.80 (d, *J* = 12.9 Hz, 1H, CH-1), 2.63 (s, 4H, morpholine), 2.37 (t, *J* = 3.0 Hz, 1H, CH-9), 2.19 (d, *J* = 13.5 Hz, 1H, CH-16), 2.05 – 1.01 (m, 17H), 1.37 (s, 3H, $CH_3$-27), 1.27 (s, 3H, $CH_3$-25), 1.16 (s, 3H, $CH_3$-26), 1.13 (s, 3H, $CH_3$-29), 0.87 (s, 3H, $CH_3$-23/24), 0.83 (s, 3H, $CH_3$-28), 0.80 (s, 1H, CH-5); $^{13}$C NMR (101 MHz, Chloroform-*d*) δ 200.25 (C11), 181.03 (C30), 169.70 (C13), 169.51 (acetoxy, C=O), 128.40 (C12), 81.09 (C3), 66.67 (morpholine C×2), 61.64 (C9), 59.53 (acetoxy, $CH_2$), 54.93 (C5), 53.14 (morpholine C×2), 48.25 (C18), 45.43 (C14), 43.75 (C20), 43.19

(C8), 40.90 (C19), 38.68 (C1), 38.07 (C4), 37.70 (C22), 36.89 (C10), 32.65 (C7), 31.86 (C17), 30.93 (C21), 28.55 (C29), 28.44 (C28), 28.13 (C23), 26.46 (C2), 26.37 (C15), 23.66 (C16), 23.36 (C27), 18.66 (C26), 17.36 (C6), 16.78 (C25), 16.42 (C24); HRMS (*m*/*z*): $[M + Na]^+$ calcd. For $C_{36}H_{56}NO_6$: 598.4108, found: 598.4150.

*tert-Butyl 4-(3β-(2-morpholinoacetoxy)-11-oxo-18β-olean-12-en-30-carbonyl)-piperazine-1-carboxylate* (**14**)

Compound **13** (0.60 g, 1.0 mmol) was dissolved in acetonitrile (20 mL), then EDCI (0.23 g, 1.2 mmol), triethylamine (0.13 g, 1.2 mmol) and HOBt (0.16 g, 1.2 mmol) were added.The mixture was stirred at room temperature for 20 min. The 1-Boc-piperazine (0.46 g, 2.5 mmol) was added, and the mixture was stirred under reflux for 10 h. The solvent was removed under vacuum to give a residue which was treated with a mixture of ethanol and water. The solution was stirred at room temperature for 30 min, and a solid was obtained by filtration while washing with $H_2O$.

A white solid; yield, 93.9%; m.p. 204.3-205.3 °C; $^1H$ NMR (400 MHz, Chloroform-*d*) δ 5.72 (t, *J* = 3.4 Hz, 1H, CH-12), 4.69 – 4.58 (m, 1H, CH-3), 3.79 (dt, *J* = 6.5, 3.3 Hz, 4H, morpholine $CH_2$×2), 3.72 – 3.59 (m, 4H, piperazine $CH_2$×2), 3.45 (d, *J* = 5.4 Hz, 4H, piperazine $CH_2$×2), 3.25 (q, *J* = 2.2 Hz, 2H, Morpholinoacetoxy $CH_2$), 2.84 (d, *J* = 13.6 Hz, 1H, CH-1), 2.64 (q, *J* = 5.1, 4.6 Hz, 4H, morpholine $CH_2$×2), 2.39 (d, *J* = 3.3 Hz, 1H, CH-9), 2.33 (d, *J* = 13.5 Hz, 1H, CH-16), 1.56 (s, 9H, tert-butyl $CH_3$×3),1.36 (s, 3H, $CH_3$-27),

1.27 (s, 3H, $CH_3$-25), 1.19 (s, 3H, $CH_3$-26), 1.15 (s, 3H, $CH_3$-29), 0.91 (s, 6H, $CH_3$-23/24), 0.84 (s, 3H, $CH_3$-28), 0.82(m, 1H, CH-5); $^{13}$C NMR (101 MHz, Chloroform-*d*) δ 199.88 (C11), 174.10 (C30), 169.96 (acetyloxy C=O), 169.49 (C13), 154.51 (Boc C=O), 128.49 (C12), 81.01 (tert-butyl C), 80.23 (C3), 66.80 (morpholine C×2), 61.63 (C9), 59.75 ( acetoxy $CH_2$), 54.93 (C5), 53.27 **(**morpholine C×2), 48.10 (C18), 45.26 (C14), 43.86 (piperazine C×2), 43.78 (C20), 43.26 ( C8/19), 38.71(C1/C4), 38.04 (piperazine C×2), 37.70 (C22), 36.88 (C10), 33.18 (C7), 32.70 (C17), 31.75 (C21), 28.40 (C29), 28.35 (tert-butyl $CH_3$×3), 28.12 (C28), 27.05 (C23), 26.67 (C2), 26.38 (C15), 23.65 (C16), 23.09 (C27), 18.65 (C26), 17.35 (C6), 16.75 (C25), 16.40 (C24); HRMS (*m*/*z*): $[M + H]^+$ calcd. for $C_{45}H_{72}N_3O_7$: 766.5370, found: 766.5301.

*4-(3β-(2-Chloroacetoxy)-11-oxo-18β-olean-12-en-30-carbonyl)piperazine-1-carboxylic acid* (**15**)

Chloroacetic anhydride (1.37 g, 8.0 mmol) and $K_2CO_3$ (2.76 g, 20.0 mmol) was heated at 130 °C in toluene for 20 min with a constant water separator. Then, compound **9** (0.64 g, 1.0 mmol) was added to the hot stirred suspension and the reaction mixture was stirred for another 1 h at 130 °C. On removal of the toluene, the $H_2O$ (50 ml) was added to the cool residue, After stirring for 1 h at room temperature, the white product was filtered, washed with cold $H_2O$ and dried.

A white solid; yield, 96.3%; m.p.205.7.0-206.6 °C; $^1$H NMR (400 MHz, Chloroform-*d*) δ 5.66 (s, 1H, CH-12), 4.59 (dd, *J* = 11.8, 4.7 Hz, 1H, CH-3), 4.15 – 3.97 (m, 4H, piperazine $CH_2$×2), 3.65 (s, 4H, piperazine $CH_2$×2), 3.52 (s, 2H,

$CH_2$-Cl), 2.85 – 2.75 (m, 1H, CH-1), 2.34 (s, 1H, CH-9), 2.28 (d, *J* = 12.1 Hz, 1H, CH-16), 1.34 (m, 3H, $CH_3$-27), 1.22 (s, 3H, $CH_3$-25), 1.14 (s, 3H, $CH_3$-26), 1.10 (s, 3H, $CH_3$-29), 0.88 (s, 6H, $CH_3$-23/24), 0.80 (s, 3H, $CH_3$-28), 0.78 (m, 1H, CH-5); $^{13}$C NMR (101 MHz, chloroform-*d*) δ 199.83 (C11), 174.27 (C30), 169.43 (C13), 167.13 (chloroacetoxy C=O), 165.34 (-COOH), 128.51 (C12), 82.99 (C3), 61.62 (C9), 54.95 (C5), 48.06 (C18), 46.28 (C14), 45.26 (piperazine C), 43.93 (piperazine C), 43.83 (C20), 43.27 (C8), 42.21 (C19), 41.23 (C1), 40.65 (C-Cl), 38.67(C4), 38.22 (piperazine C×2), 37.67(C22), 36.88 (C10), 33.01 (C7), 32.68 (C17), 31.76 (C21), 28.39 (C29), 28.00 (C28), 27.03 (C23), 26.65 (C2), 26.35 (C15), 23.40 (C16), 23.11 (C27), 18.65 (C26), 17.31 (C6), 16.60 (C25), 16.40 (C24); HRMS (*m*/*z*): [M + H] $^+$ calcd. for $C_{37}H_{56}ClN_2O_6$: 659.3827, found: 659.3873.

*4-(3β-(2-Morpholinoacetoxy)-11-oxo-18β-olean-12-en-30-carbonyl)piperazine-1-carboxylic acid* (**16**)

Compound **15** (0.66 g, 1.0 mmol), morpholine (0.13 g, 1.5 mmol), $K_2CO_3$ (0.69 g, 5.0 mmol) and a catalytic amount of $I_2$ in absolute ethanol (15 mL) was stirred under reflux for 12 h. The reaction mixture was evaporated under reduced pressure and the residue was dissolved in ethanol/$H_2O$ mixture and the white precipitate was collected by filtration.

A white solid; yield, 91.2%; m.p. 196.0-196.9 °C; $^1$H NMR (400 MHz, Chloroform-*d*) δ 5.66 (d, *J* = 2.9 Hz, 1H, CH-12), 4.58 (dd, *J* = 11.7, 4.3 Hz, 1H, CH-3), 3.70 (t, *J* = 4.5 Hz, 4H, morpholine $CH_2$×2), 3.60 (s, 8H, piperazine $CH_2$×4 ), 3.26 –

3.16 (m, 2H, morpholinoacetoxy $CH_2$), 2.86 – 2.74 (m, 1H, CH-1), 2.52 (d, *J* = 5.2 Hz, 4H, morpholine $CH_2$×2), 2.33 (s, 1H, CH-9), 2.27 (d, *J* = 13.2 Hz, 1H, CH-16),1.34 (s, 3H, $CH_3$-27), 1.21 (s, 3H, $CH_3$-25), 1.14 (s, 3H, $CH_3$-26), 1.09 (s, 3H, $CH_3$-29), 0.87 (s, 6H, $CH_3$-23/24), 0.79 (s, 3H, $CH_3$-28), 0.77(m, 1H, CH-5); $^{13}$C NMR (101 MHz, Chloroform-*d*) δ 199.84 (C11), 174.27 (C30), 169.52 (C13), 167.71 (acetyloxy C=O), 167.13 (COOH), 128.48 (C12), 82.98 (C3), 66.76 (morpholine C×2), 61.62 (C9), 61.45 (acetoxy $CH_2$), 54.95 (C5), 53.44 (morpholine C×2), 48.10 (C18), 45.26 (C14), 43.91 (piperazine C×2), 43.79 (C20), 43.27 ( C8), 41.86 (19), 41.23 (C1), 38.66 (C4), 38.22 (piperazine C×2), 37.68 (C22), 36.87 (C10), 33.09 (C7), 32.67 (C17), 31.76 (C21), 28.40 (C29), 28.00 (C28), 27.04 (C23), 26.66 (C2), 26.36 (C15), 23.40 (C16), 23.11 (C27), 18.64 (C26), 17.31 (C6), 16.60 (C25), 16.40 (C24); HRMS (Methanol as solvent, *m*/*z*): $[M_{Methyl\ ester} + H]^+$ calcd. for $C_{42}H_{66}N_3O_7$: 724.4901, found: 724.9726.

*3β-(2-Morpholinoacetoxy)-11-oxo-18β-olean-12-en-30-carbonyl piperazine* (**17**)

Compound **14** (0.77g, 1.0 mmol) or compound **16** (0.71 g, 1.0 mmol) was dissolved in $CH_2Cl_2$ (10 mL) at 0 °C under stirring. Trifluoroacetic acid (5 mL) was added, and the reaction was stirred at 0 °C for 3 h. After reaction, the mixture was made basic with a saturated $Na_2CO_3$ solution. This mixture was extracted with $CH_2Cl_2$, dried over $Na_2SO_4$, and concentrated to give the desired product. A white solid; yield, 91.7%; m.p. 215.9-216.7 °C; $^1$H NMR (400 MHz, Chloroform-*d*) δ 5.70 (t, *J* = 3.2 Hz, 1H, CH-12), 4.60 (dt, *J* = 11.2, 4.7 Hz, 1H, CH-3), 3.76 (dt, *J* = 6.7, 3.3 Hz, 4H, morpholine $CH_2$×2), 3.62 (t, *J* = 5.5 Hz, 4H, piperazine

$CH_2$×2), 3.21 (dd, *J* = 4.5, 2.4 Hz, 2H, morpholinoacetoxy $CH_2$), 2.91 – 2.84 (m, 4H, piperazine $CH_2$×2), 2.81 (d, *J* = 13.0 Hz, 1H, CH-1), 2.60 (t, *J* = 5.2 Hz, 4H, morpholine $CH_2$×2), 2.36 (t, *J* = 3.3 Hz, 1H, CH-9), 2.30 (d, *J* = 13.5 Hz, 1H, CH-16), 1.36 (s, 3H, $CH_3$-27), 1.23 (s, 3H, $CH_3$-25), 1.16 (s, 3H, $CH_3$-26), 1.12 (s, 3H, $CH_3$-29), 0.88 (s, 6H, $CH_3$-23/24), 0.82 (s, 3H, $CH_3$-28), 0.79 (s, 1H, CH-5); $^{13}C$ NMR (101 MHz, $cdcl_3$) δ 199.95 (C11), 173.84 (C30), 170.01 (acetyloxy C=O), 169.73 (C13), 128.47 (C12), 81.03 (C3), 66.83 (morpholine C×2), 61.65 (C9), 59.78 (morpholinoacetoxy $CH_2$), 54.95 (C5), 53.30 (morpholine C×2), 48.23(C18), 46.30 (piperazine C×2), 45.28 (C14), 43.79 (C20), 43.75 (C8), 43.28 (piperazine C×2), 38.75 (C19), 38.06 (C1/4), 37.75 (C22), 36.91 (C10), 33.40 (C7), 32.71 (C17), 31.77 (C21), 28.43 (C29), 28.13 (C28), 27.05 (C23), 26.71 (C2), 26.44 (C15), 23.66 (C16), 23.11 (C27), 18.67 (C26), 17.37 (C6), 16.77 (C25), 16.42 (C24) ; HRMS (*m*/*z*): $[M + H]^+$ calcd. for $C40H_{64}N_3O_5$: 666.4846, found: 666.4795.

*3β-Acetoxy-30-(4-(3-fluorobenzoyl)-1-piperazinyl)-olean-12-ene-11,30-dione* (**18**)

Compound **4** (0.58 g, 1.0 mmol) and triethylamine (0.13 g, 1.2 mmol) were dissolved in $CH_2Cl_2$ (20 mL) at 0 °C under stirring. 3-fluorobenzoyl chloride (0.158 g, 1.0 mmol) was added, and the reaction was stirred at room temperature for 3 h. After reaction, the mixture was washed twice with water. The organic layer was dried over anhydrous Na2SO4, filtered, and concentrated under reduced pressure. The crude product was then chromatographed on silica

(DCM-methanol, 20:1).

A white solid; yield, 88.2%; m.p. 234.1-225.9 °C; $^1$H NMR (400 MHz, Chloroform-*d*) δ 7.41 (td, *J* = 7.8, 5.6 Hz, 1H, phenyl), 7.23 – 7.09 (m, 3H, phenyl), 5.68 (s, 1H, CH-12), 4.52 (dd, *J* = 11.6, 4.7 Hz, 1H, CH-3), 3.71 – 3.45 (m, 8H, morpholine $CH_2$×4), 2.79 (dt, *J* = 13.5, 3.6 Hz, 1H, CH-1), 2.35 (s, 1H, CH-9), 2.32 – 2.23 (m, 1H, CH-16), 2.05 (s, 3H, acetyloxy $CH_3$), 1.35 (s, 3H, $CH_3$-27), 1.25 (s, 3H, $CH_3$-25), 1.24 (s, 3H, $CH_3$-26), 1.16 (s, 3H, $CH_3$-29), 1.12 (s, 3H, $CH_3$-23), 0.88 (s, 3H, $CH_3$-24), 0.82 (s, 3H, $CH_3$-28), 0.79 (m, 1H, CH-5); $^{13}$C NMR (101 MHz, Chloroform-*d*) δ 199.92 (C11), 174.25 (C30) – acetyloxy C=O), 169.31 (C13), 169.06 (benzoyl), 169.04 (benzoyl), 163.78 (C-F), 161.31 (C-F), 137.17 (phenyl), 137.10 (phenyl), 130.52 (phenyl), 130.44 (phenyl), 128.53 (C12), 122.73 (phenyl), 122.70 (phenyl), 117.21 (phenyl), 117.01 (phenyl), 114.56 (phenyl), 114.33 (phenyl), 80.56 (C3), 61.69 (C9), 55.00 (C5), – (C18), 45.28 (C14), 43.91 (C20), 43.70 (morpholine), 43.26 ( C8), 38.78 (C19), 38.02 (C1/4), 37.67 (C22), 36.91 (C10) – (C7), 32.72 (C17), 31.78 (C21), 28.39 (C29), 28.02 (C28), 27.04 (C23), 26.66 (C2), 26.37 (C15), 23.54 (C16), 23.08 (C27), 21.32 (acetoxy $CH_3$), 18.65 (C26), 17.35 (C6), 16.67 (C25), 16.41 (C24); HRMS (*m*/*z*): $[M + H]^+$ calcd. for $C_{43}H_{60}FN_2O_5$: 703.4486, found: 703.4486.

*Crystal structure analysis of compound (**18**)*

The single crystal X-ray diffraction data of compound **18** was collected on a Bruker SMART APEX II CCD detector employing graphitemonochromated Cu Kα radiation (λ = 1.54178 Å) at 273 (2) K. The structures were solved by direct methods using SHELXL-97 and refined using full-matrix least-squares calculation on F2 using SHELXL-97. All non-hydrogen atoms were refined anisotropically. The hydrogen atom positions were geometrically idealized and allowed to ride on their parent atoms. Crystallographic data for compound **18** has been deposited at the Cambridge Crystallographic Data Centre. CCDC 1904891. These data can be obtained free of charge from The Cambridge Crystallographic Data Centre via www.ccdc.cam.ac.uk/data_request/cif.

*3β-Acetoxy-11-oxo-18β-olean-12-en-30-oic acid* (**2**)

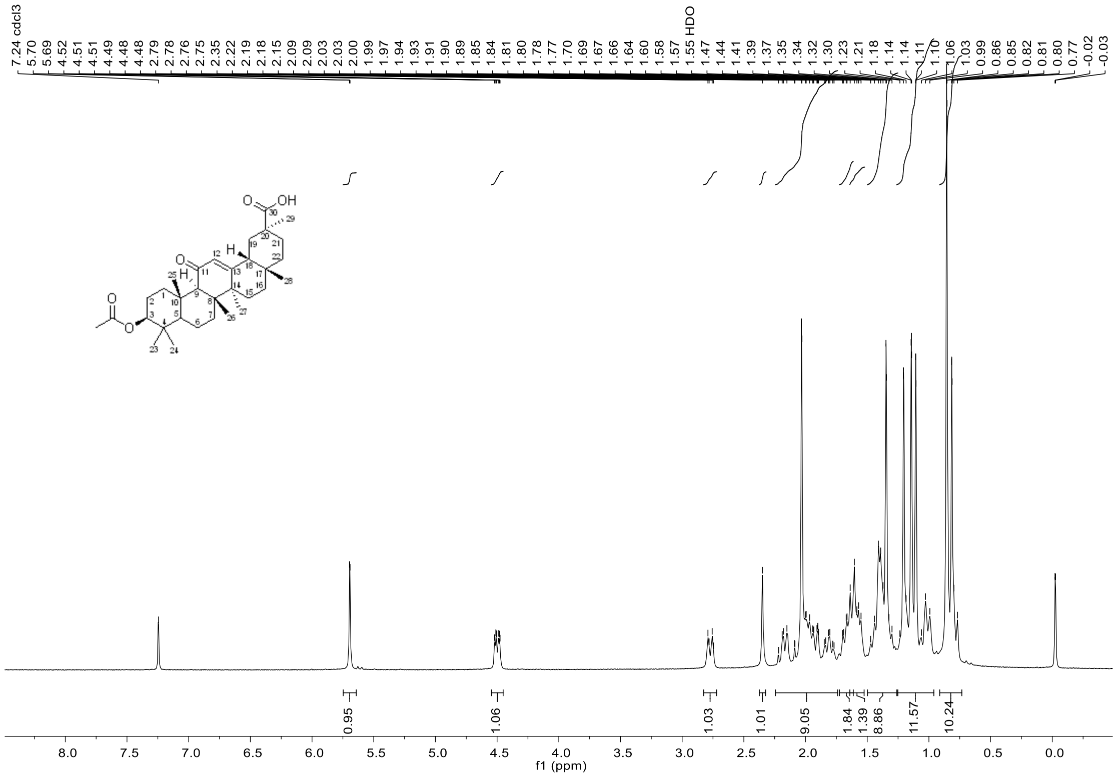


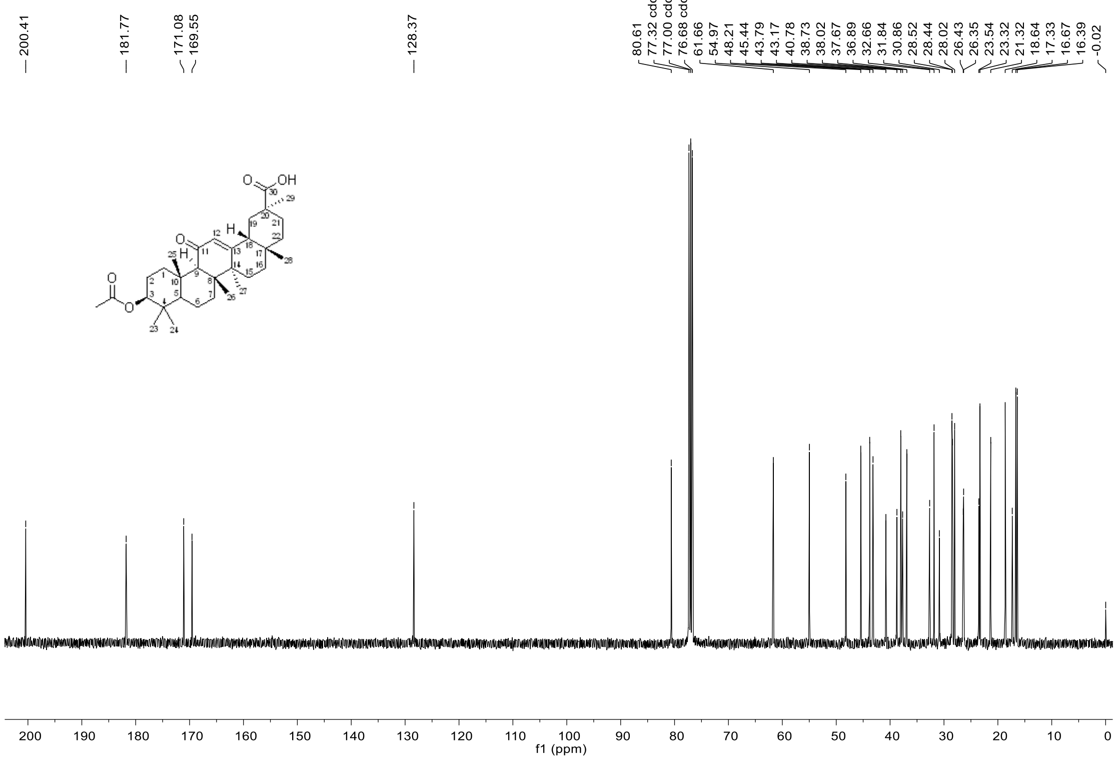

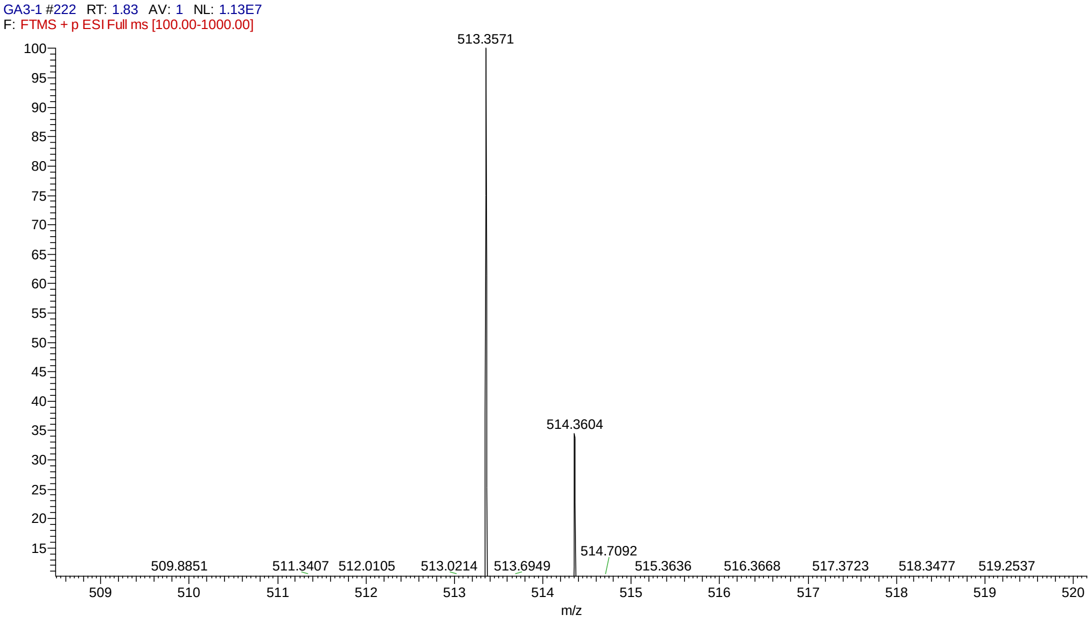


*tert-Butyl 4-(3β-acetoxy-11-oxo-18β-olean-12-en-30-carbonyl)piperazine-1-carboxylate* (**8**)

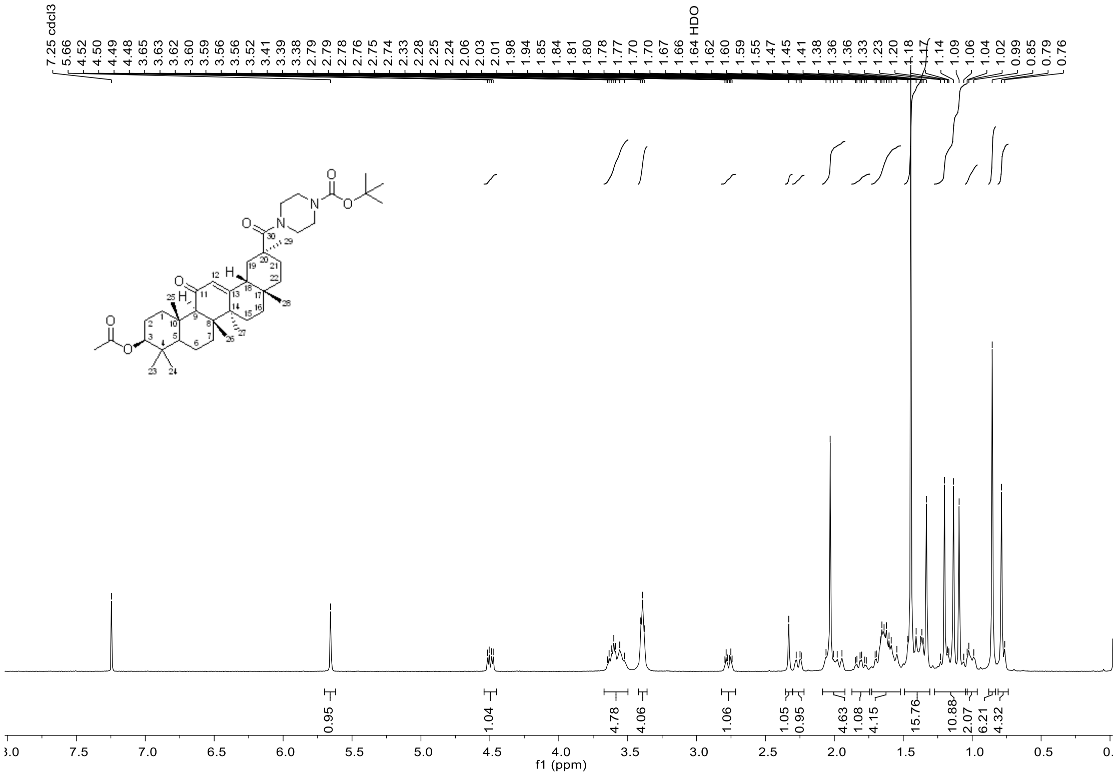

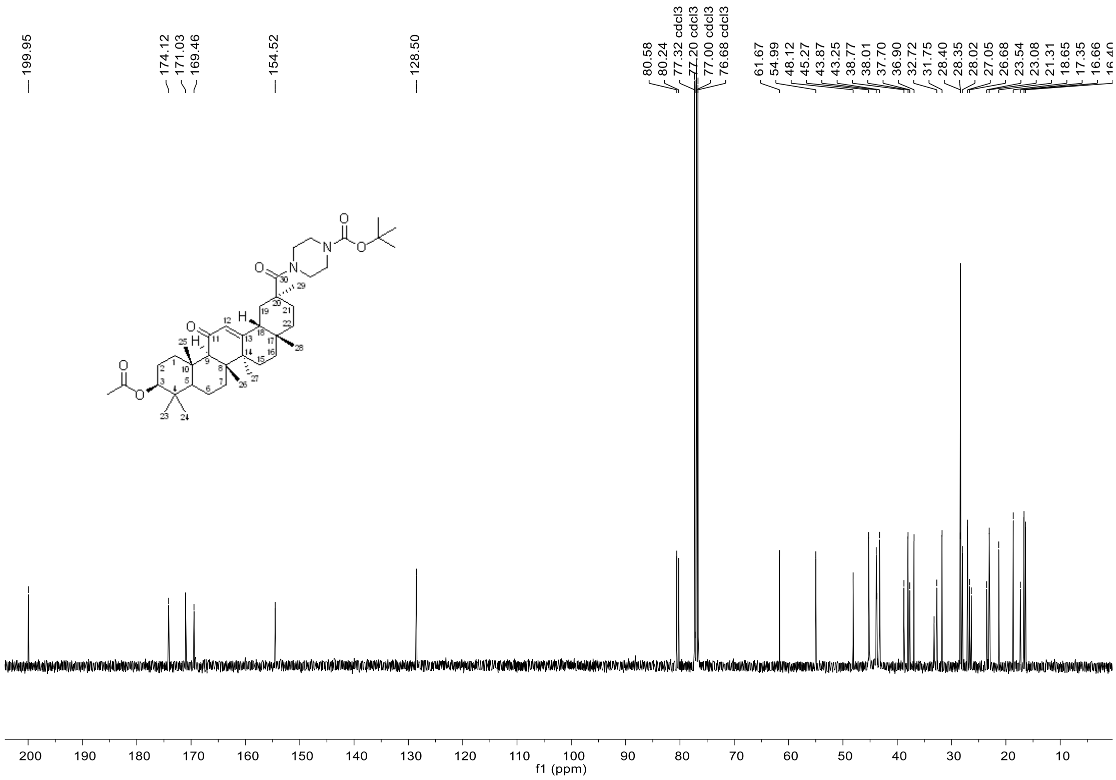
199.95
174.12
171.03
169.46
154.52
128.50
80.58
80.24
77.32 cdcl3
77.20 cdcl3
77.00 cdcl3
76.68 cdcl3
61.67
54.99
48.12
45.27
43.87
43.25
38.77
38.01
37.70
36.90
32.72
31.75
28.40
28.35
28.02
27.05
26.68
23.54
23.08
21.31
18.65
17.35
16.66
f1 (ppm)

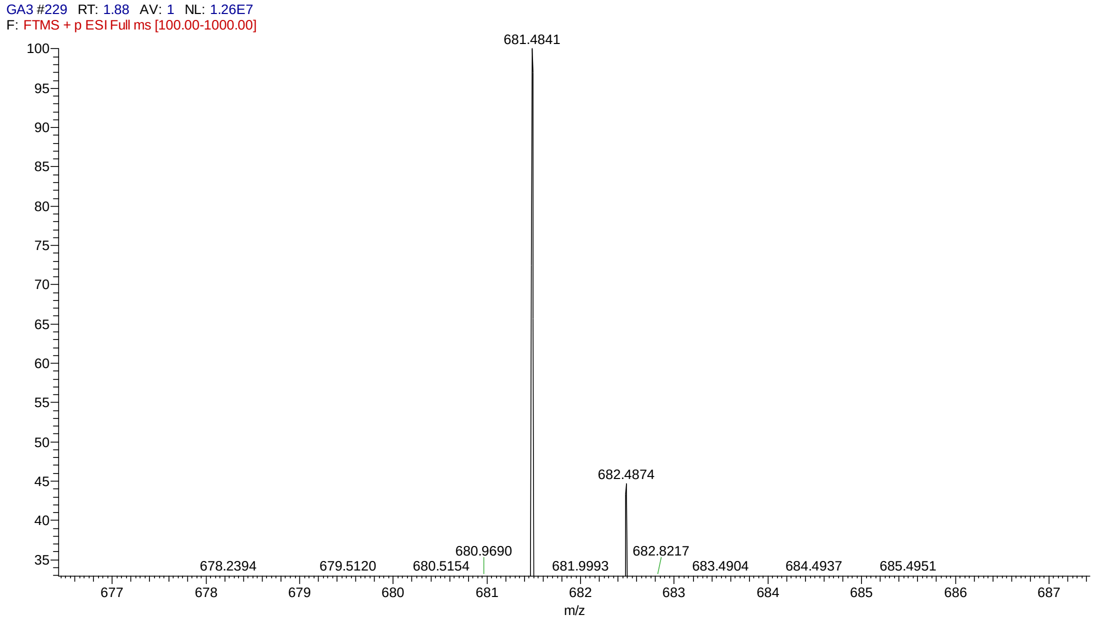
GA3 #229 RT: 1.88 AV: 1 NL: 1.26E7
F: FTMS + p ESI Full ms [100.00-1000.00]
681.4841
682.4874
680.9690
682.8217
678.2394
679.5120
680.5154
681.9993
683.4904
684.4937
685.4951
m/z

*3β-(2-Chloroacetoxy)-11-oxo-18β-olean-12-en-30-oic acid* (**12**)

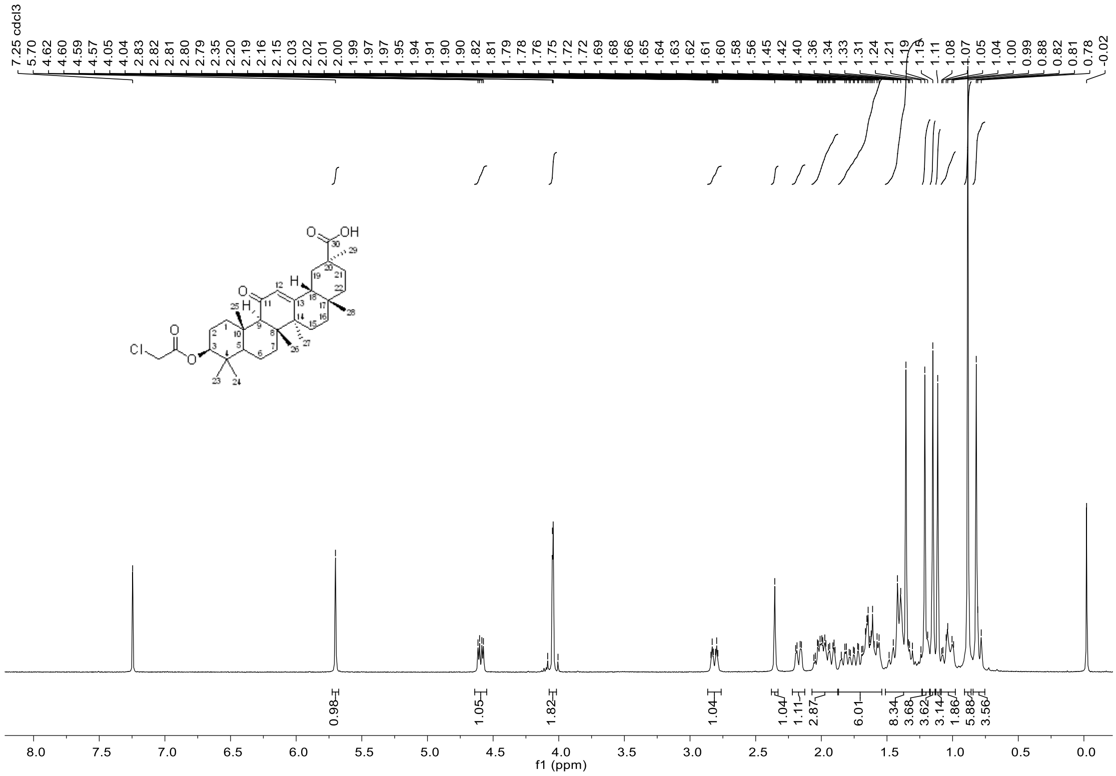

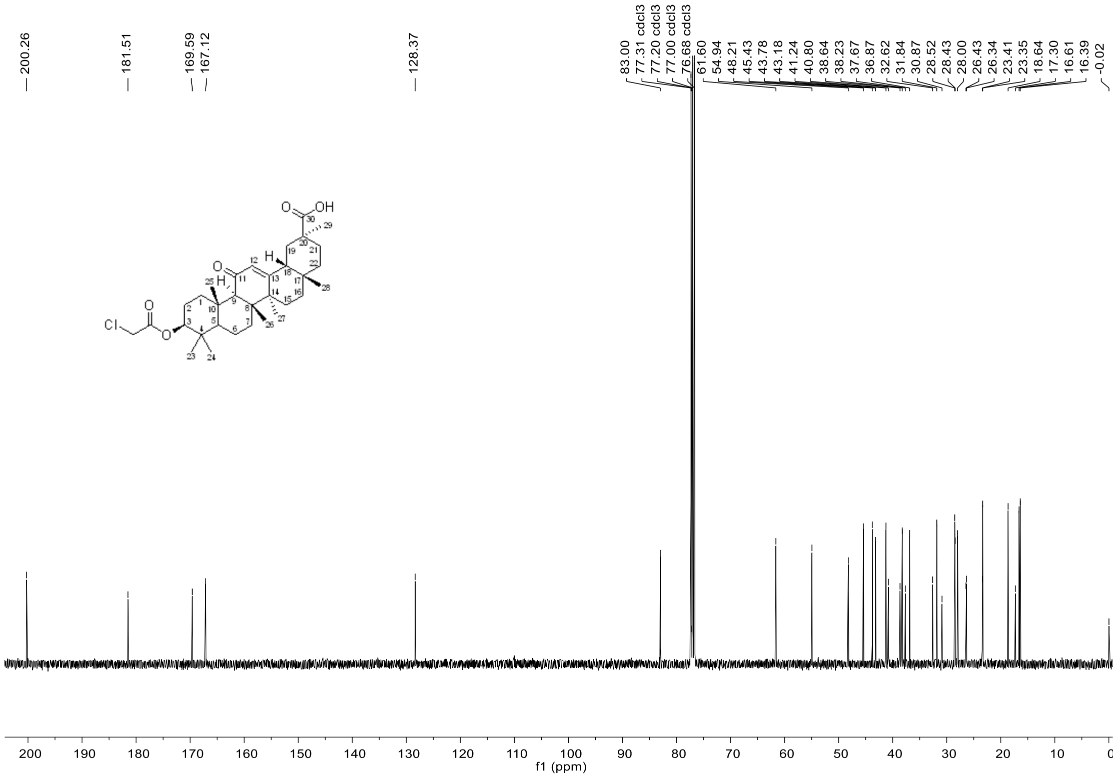

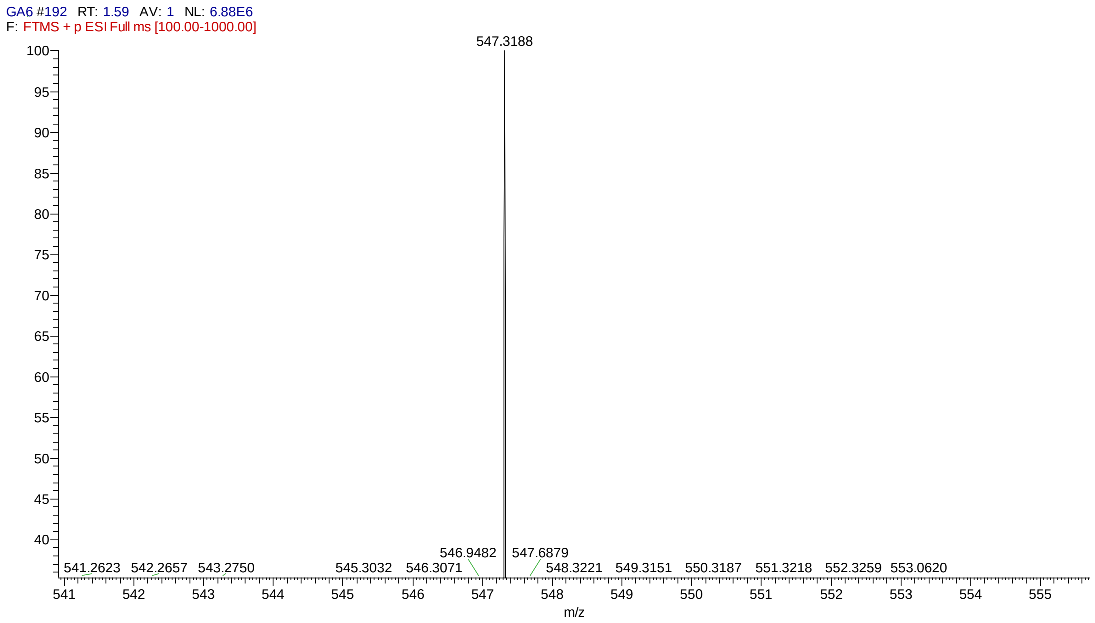


*1H-Benzo[d][1,2,3]triazol-1-yl-3β-acetoxy-11-oxo-olean-12-en-30-oate* (**6**)

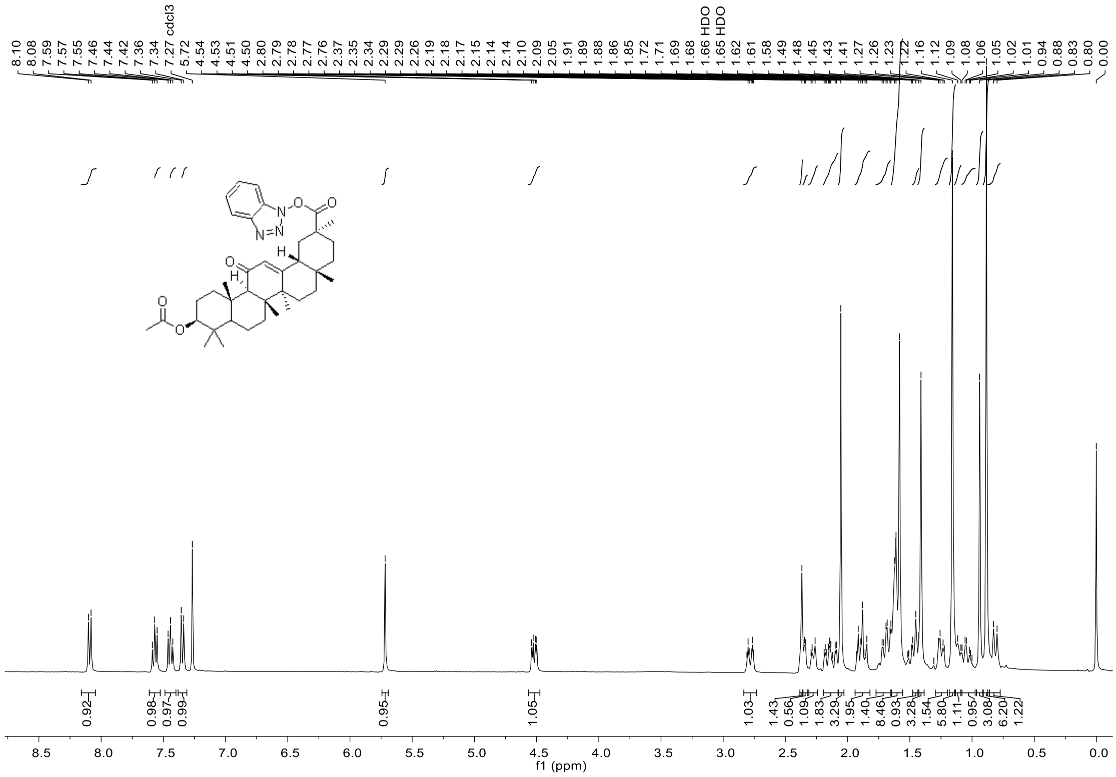

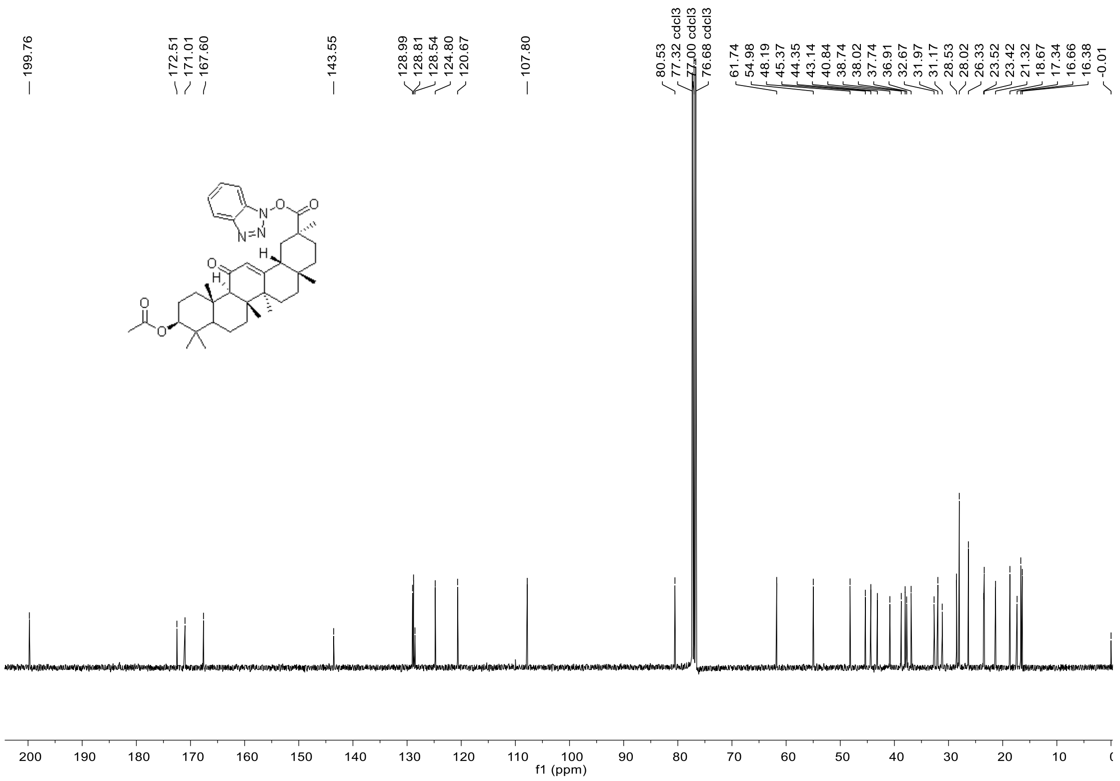

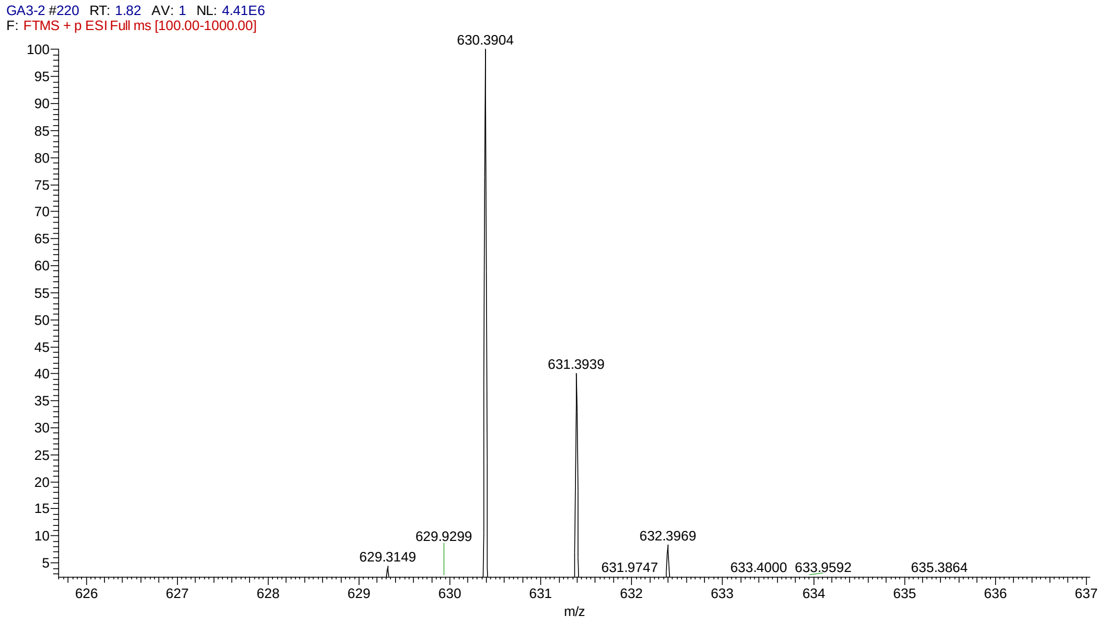
GA3-2 #220 RT: 1.82 AV: 1 NL: 4.41E6
F: FTMS + p ESI Full ms [100.00-1000.00]
630.3904
631.3939
629.9299
629.3149
632.3969
631.9747
633.4000
633.9592
635.3864
m/z

*1H-Benzo[d][1,2,3]triazol-1-yl-3β-hydroxy-11-oxo-olean-12-en-30-oate* (**7**)

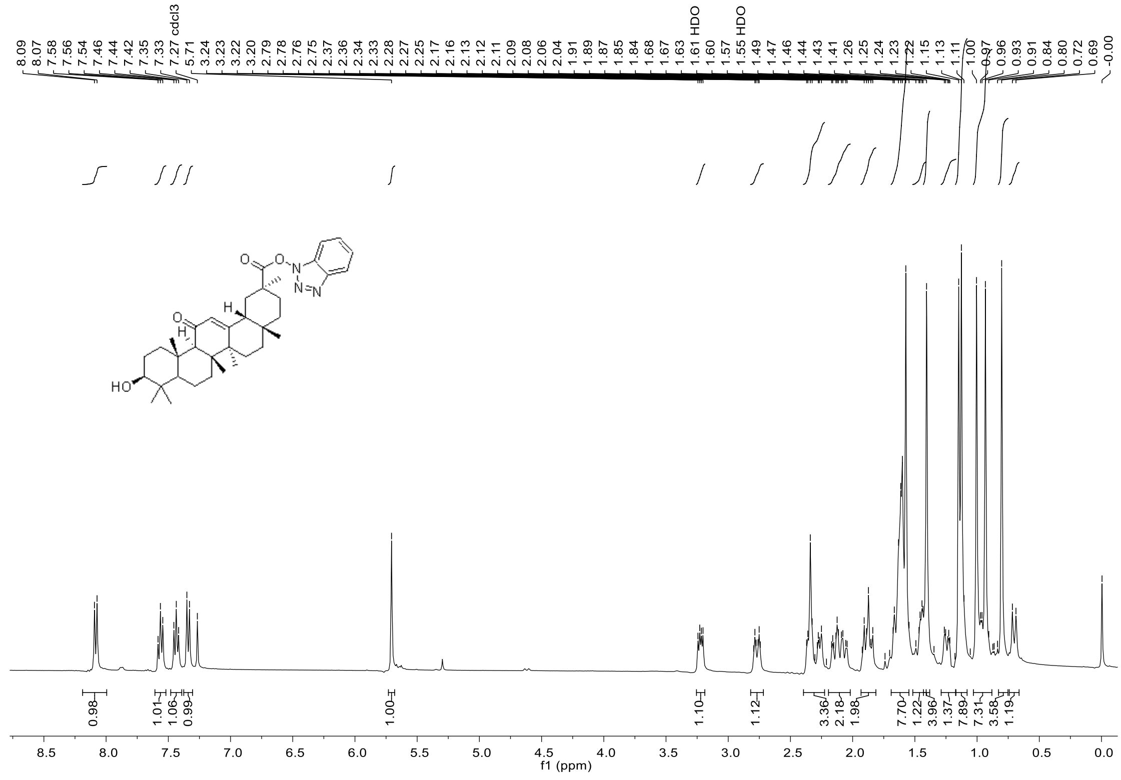


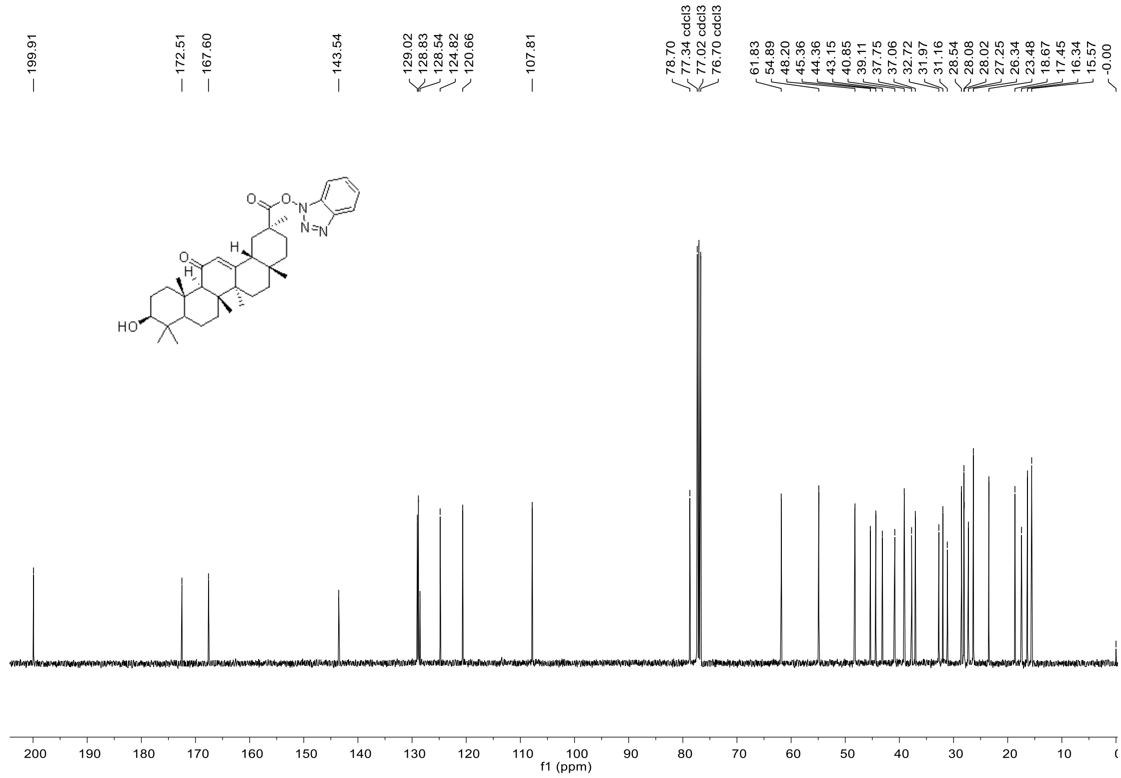

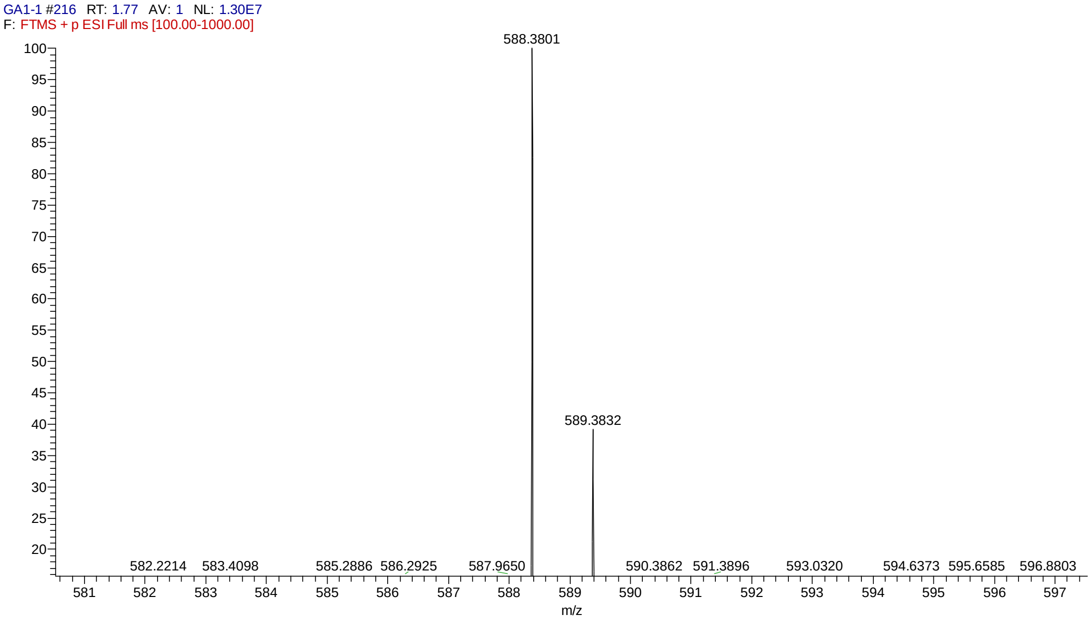


*tert-Butyl 4-(3β-hydroxyl-11-oxo-18β-olean-12-en-30-carbonyl)piperazine-1-carboxylate* (**9**)

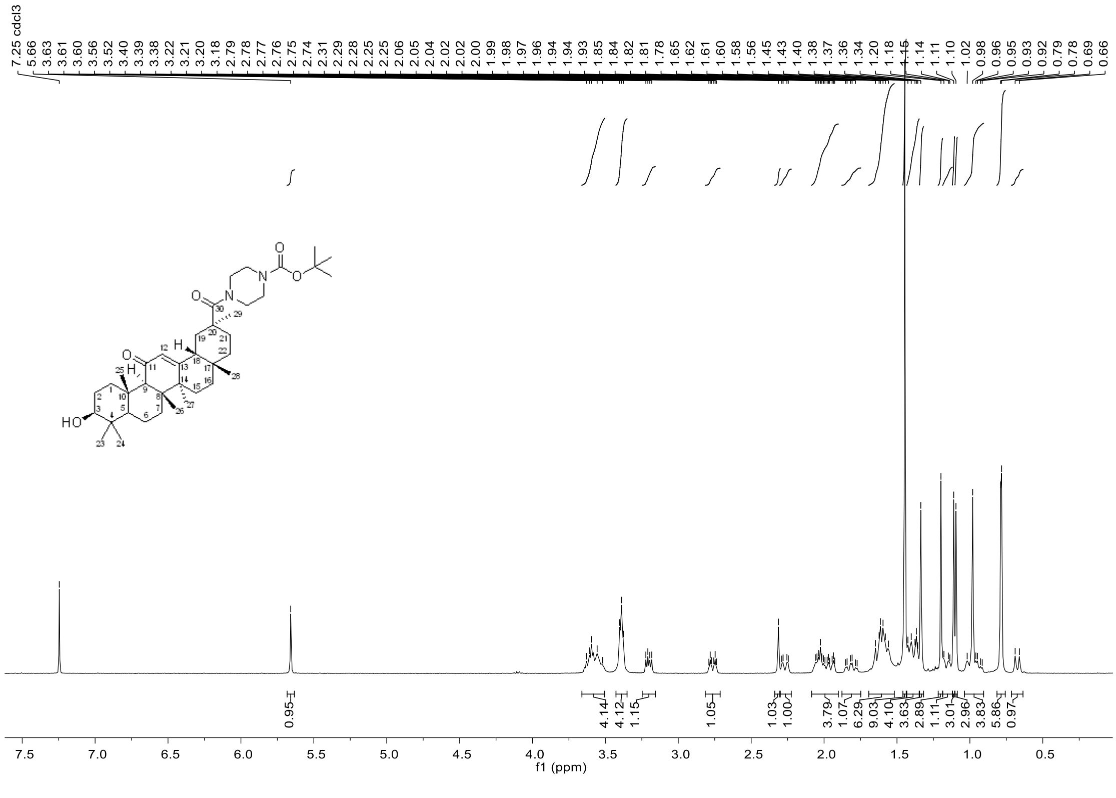

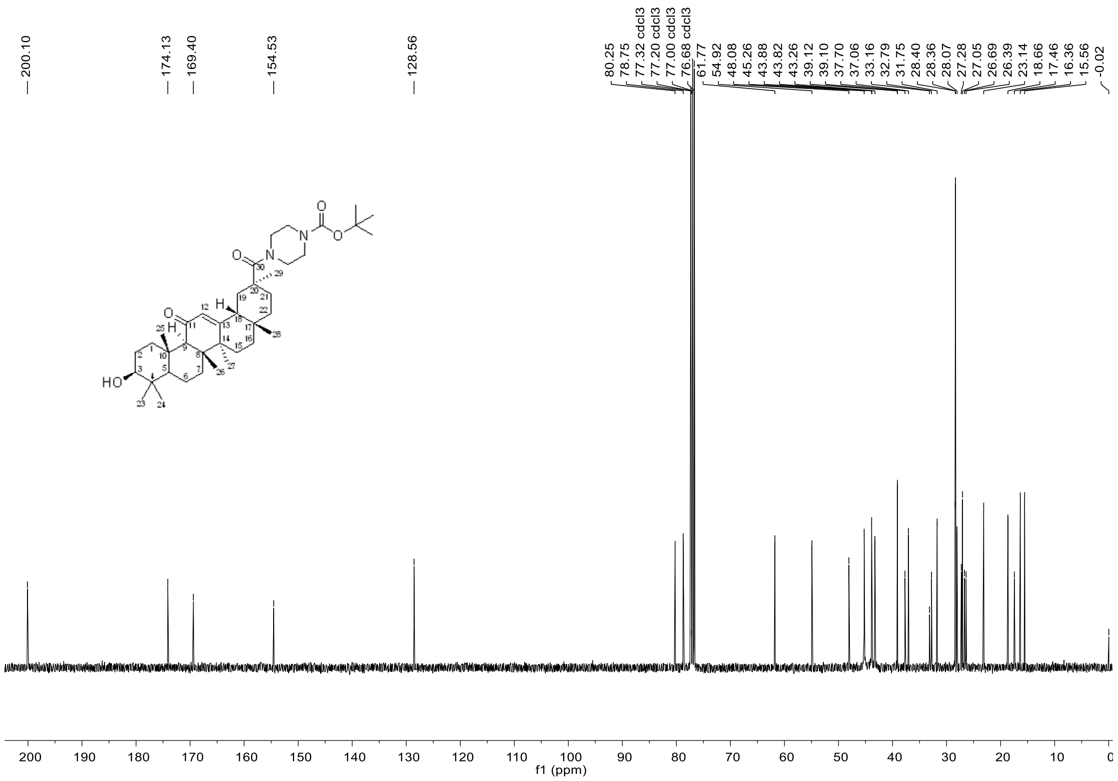
200.10
174.13
169.40
154.53
128.56
80.25
78.75
77.32 cdcl3
77.20 cdcl3
77.00 cdcl3
76.68 cdcl3
61.77
54.92
48.08
45.26
43.88
43.82
43.26
39.12
39.10
37.70
37.06
33.16
32.79
31.75
28.40
28.36
28.07
27.28
27.05
26.69
26.39
23.14
18.66
17.46
16.36
15.56
-0.02
HO
O
H
N
f1 (ppm)

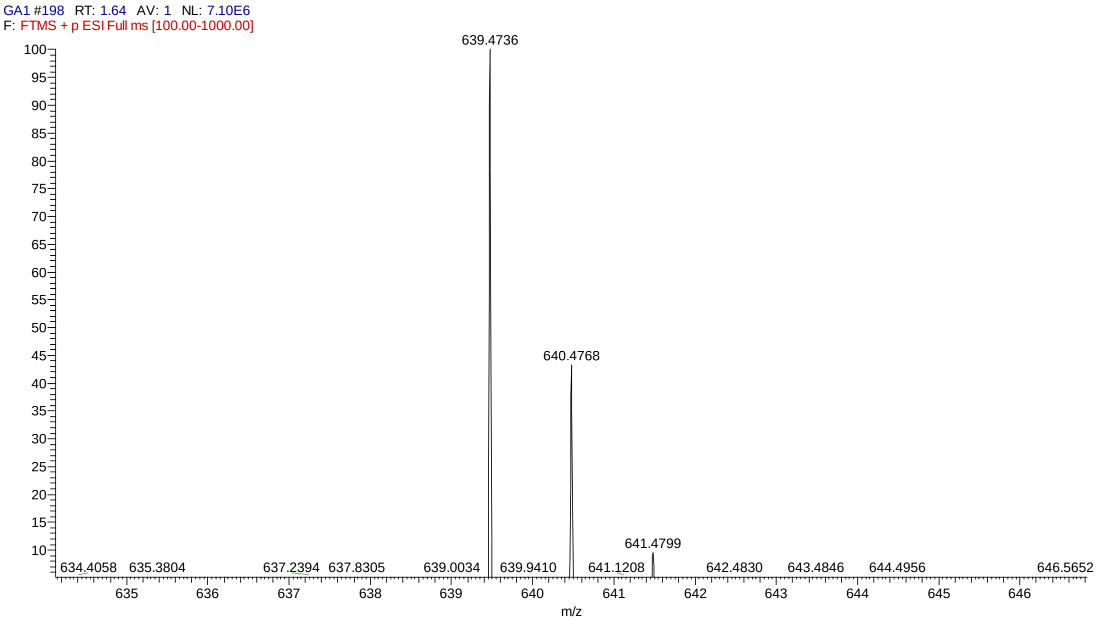
GA1 #198 RT: 1.64 AV: 1 NL: 7.10E6
F: FTMS + p ESI Full ms [100.00-1000.00]
639.4736
640.4768
641.4799
634.4058
635.3804
637.2394
637.8305
639.0034
639.9410
641.1208
642.4830
643.4846
644.4956
646.5652
m/z

*tert-Butyl 4-(3β- (2-morpholinoacetoxy)-11-oxo-18β-olean-12-en-30-carbonyl)piperazine-1-carboxylate* (**14**)

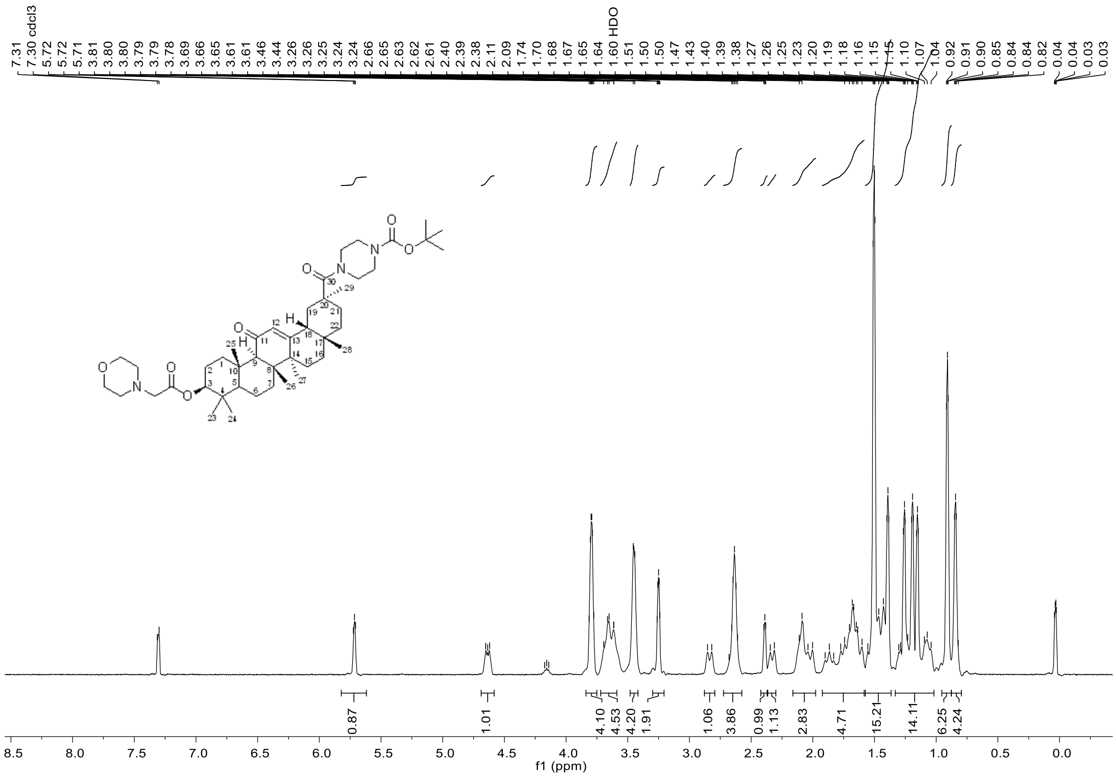

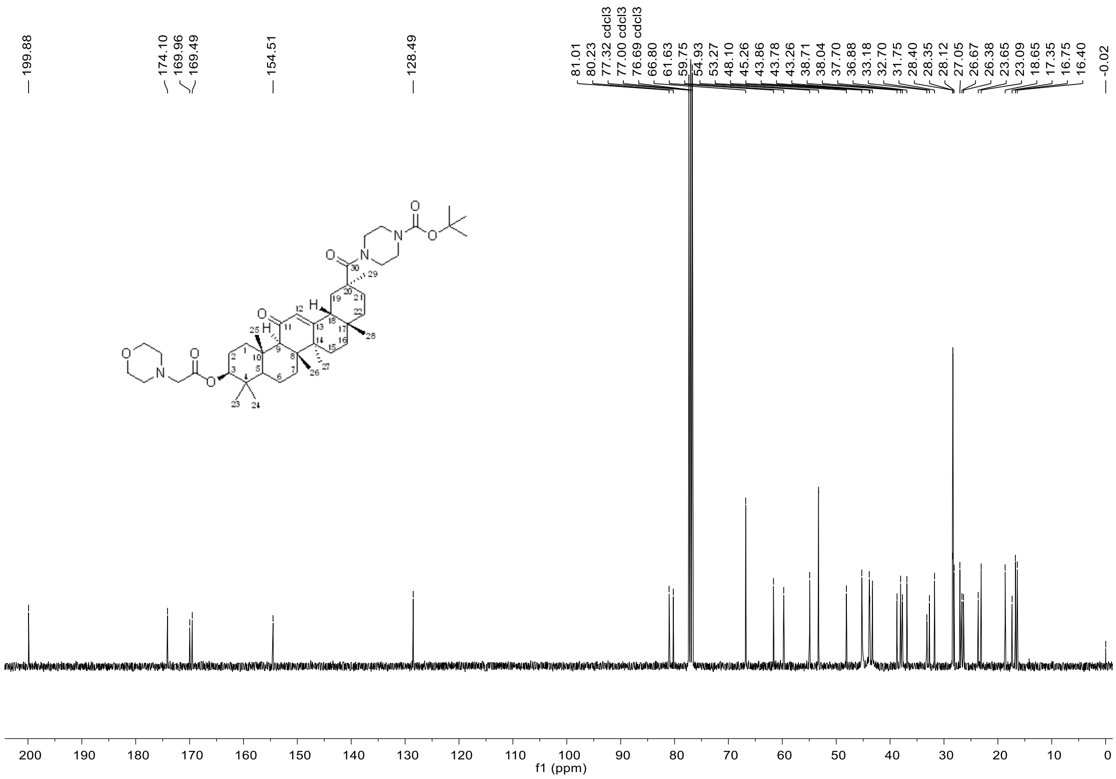

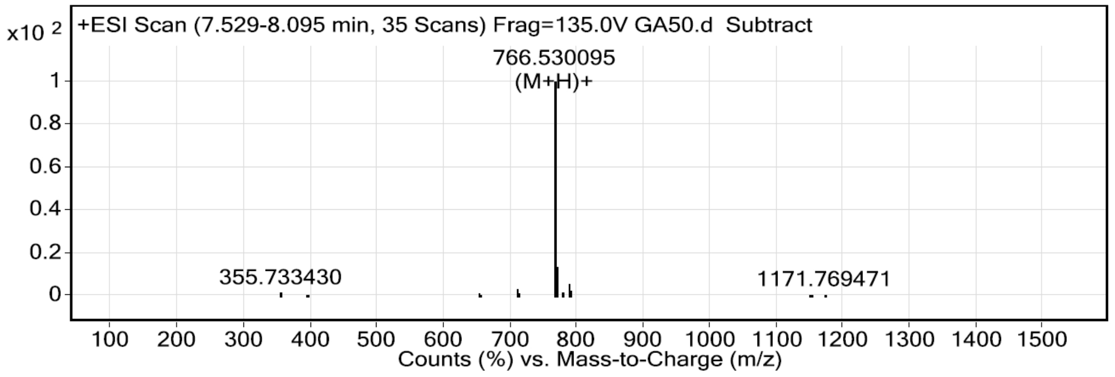


*N-(2-Chloroacetyl)piperazinyl 3β-(2-chloroacetoxy)-11-oxo-18β-olean-12-en-30-amide* (**11**)

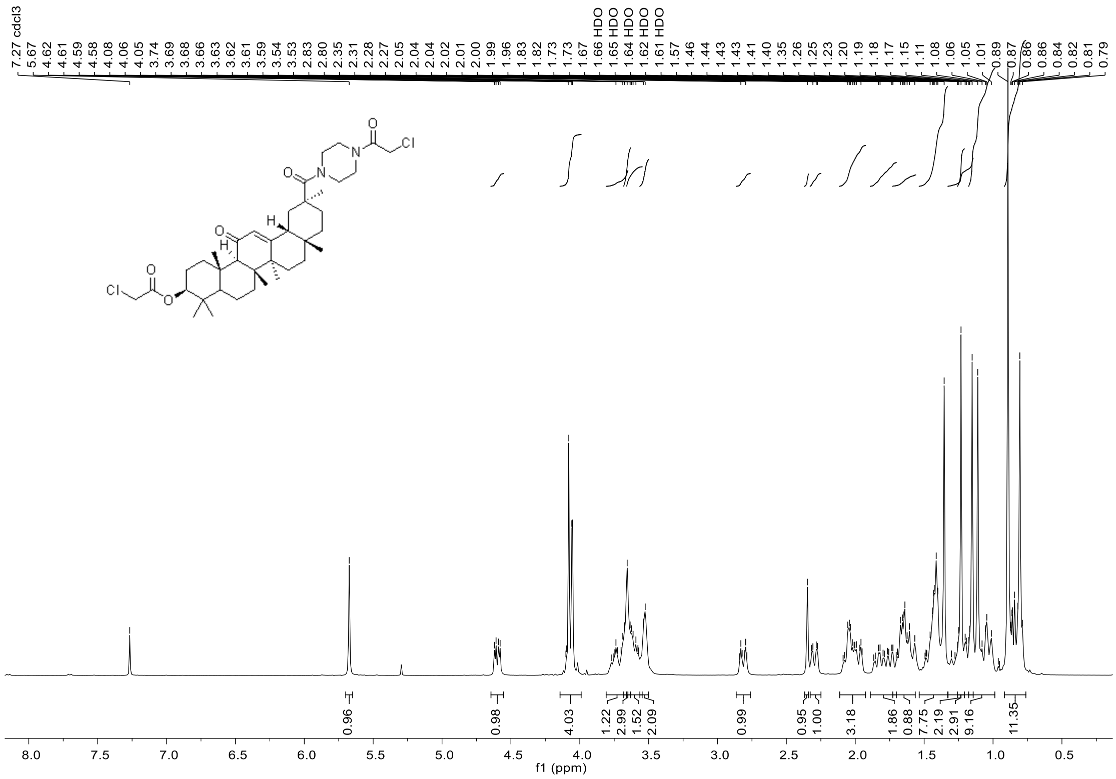

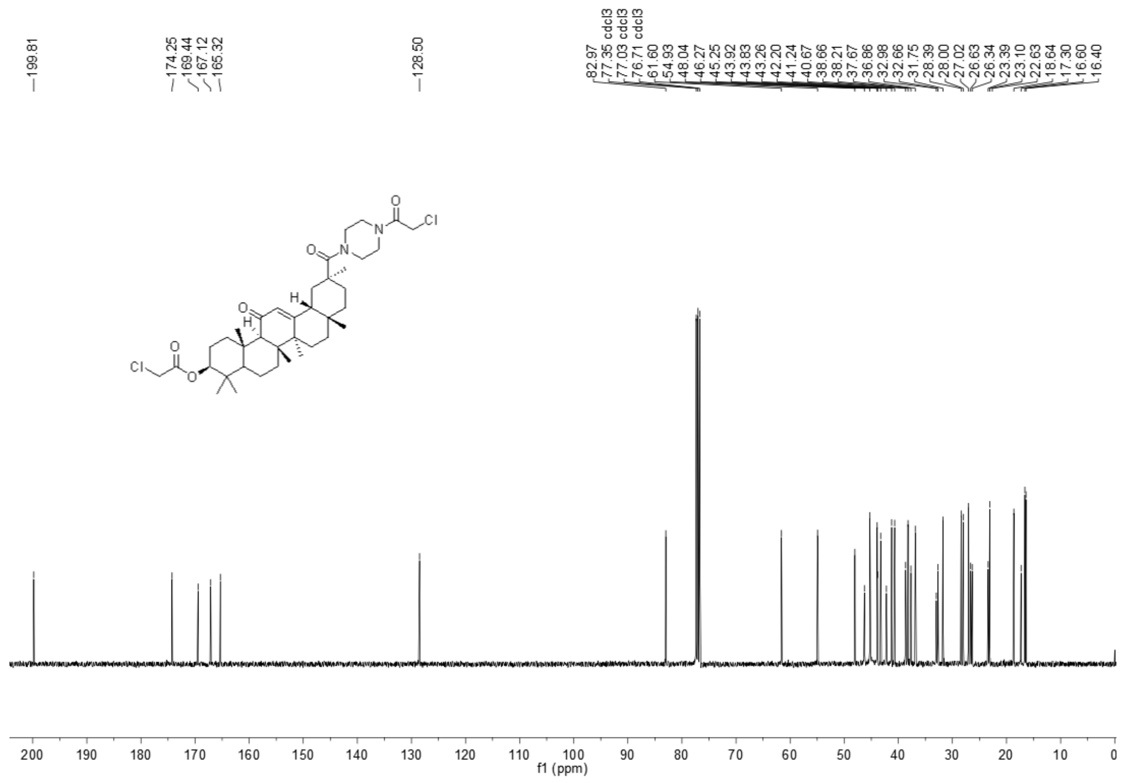
199.81
174.25
169.44
167.12
165.32
128.50
82.97
77.35 cdcl3
77.03 cdcl3
76.71 cdcl3
61.60
54.93
48.04
46.27
45.25
43.92
43.83
43.26
42.20
41.24
40.67
38.66
38.21
37.67
36.86
32.98
32.66
31.75
28.39
28.00
27.02
26.63
26.34
23.39
23.10
22.63
18.64
17.30
16.60
16.40
Cl
O
N
H
f1 (ppm)

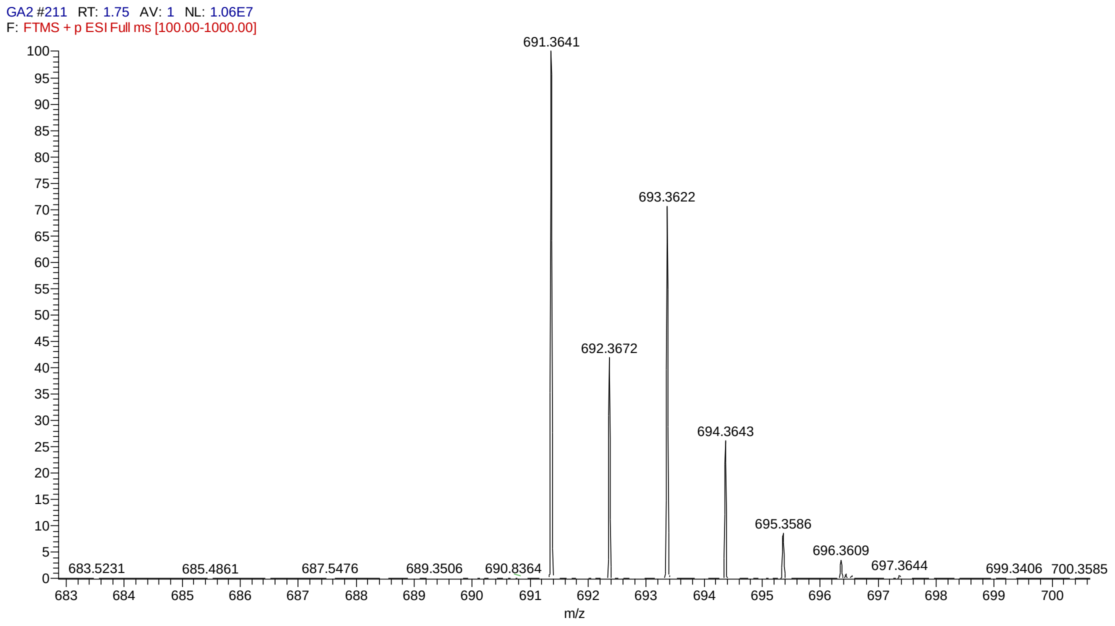
GA2 #211 RT: 1.75 AV: 1 NL: 1.06E7
F: FTMS + p ESI Full ms [100.00-1000.00]
691.3641
693.3622
692.3672
694.3643
695.3586
696.3609
697.3644
683.5231
685.4861
687.5476
689.3506
690.8364
699.3406
700.3585
m/z

*4-(3β-(2-Chloroacetoxy)-11-oxo-18β-olean-12-en-30-carbonyl)piperazine-1-carboxylic acid* (**15**)

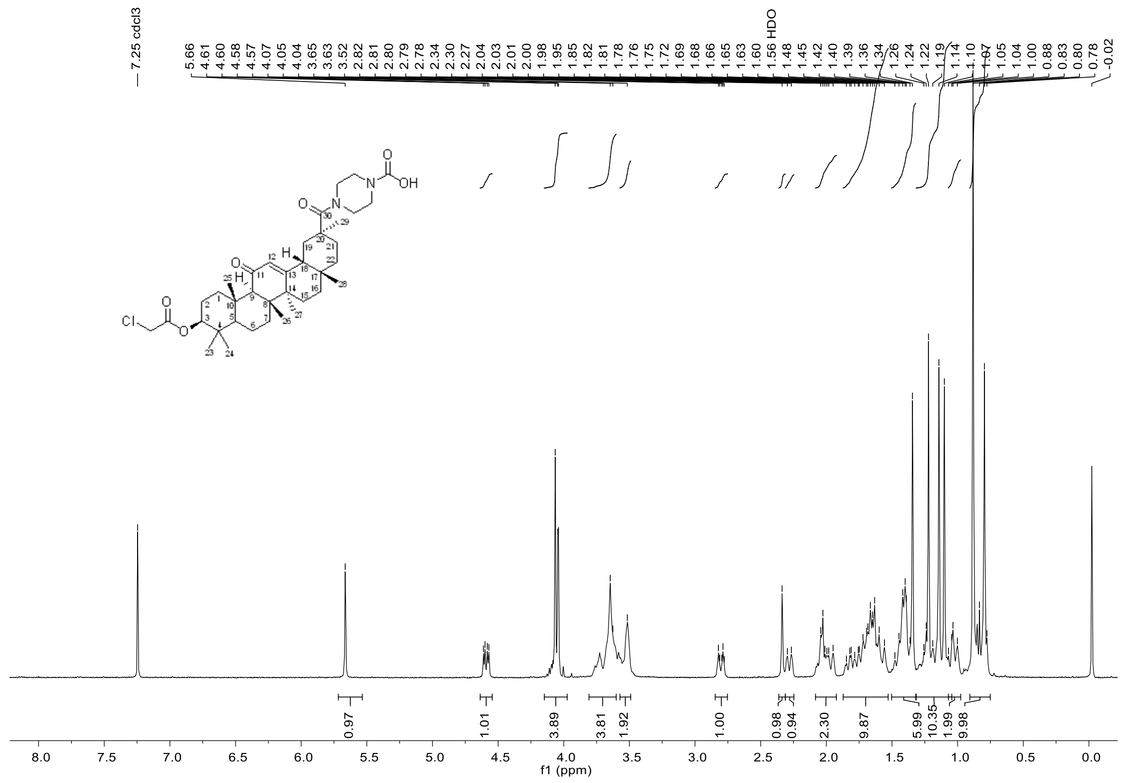

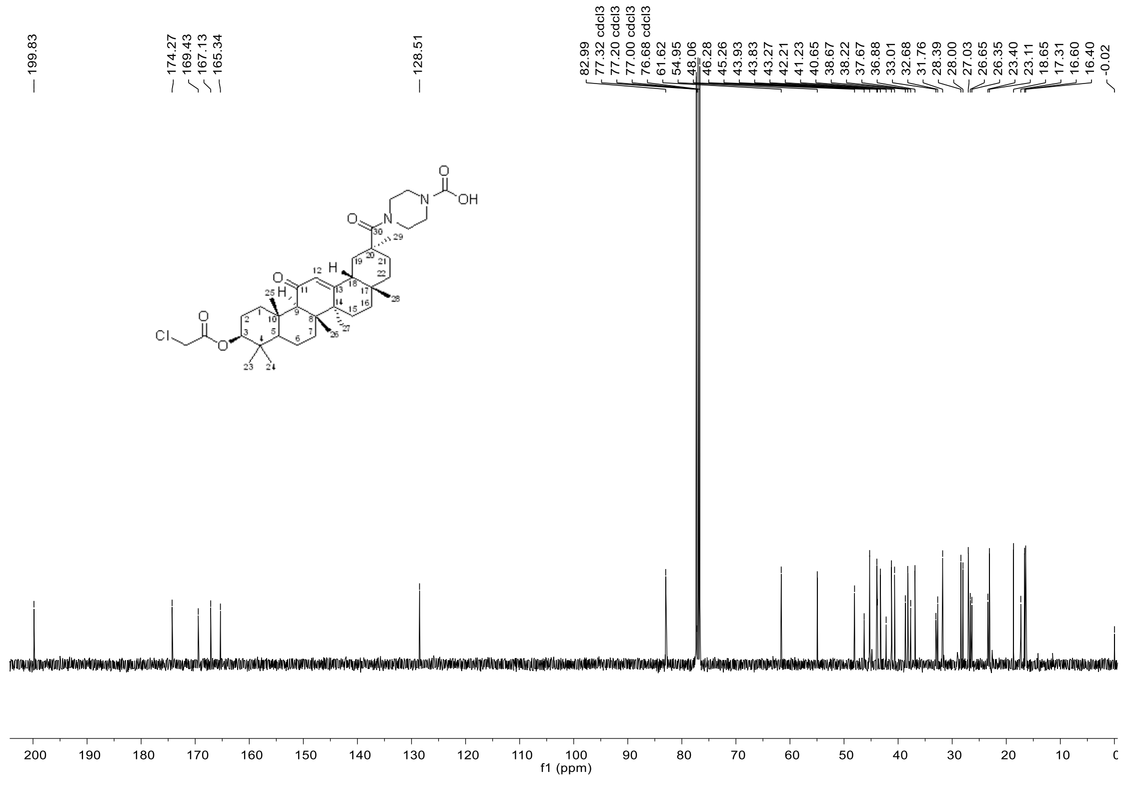

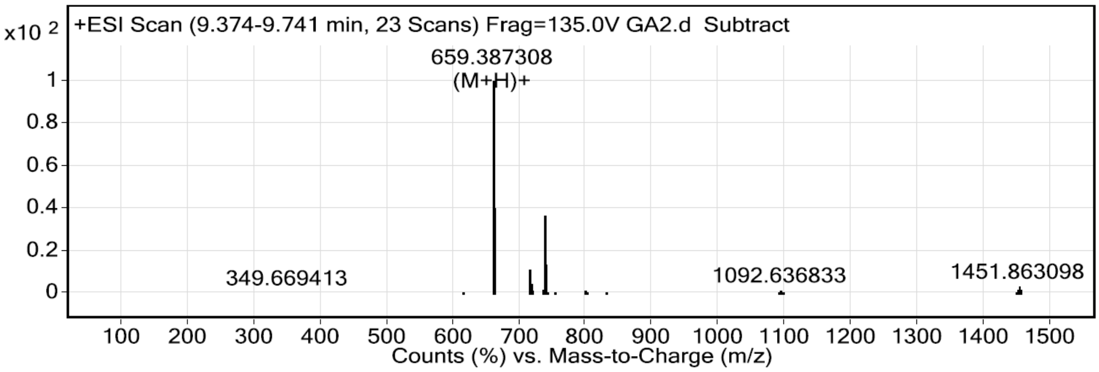


*3β-(2-Morpholinoacetoxy)-11-oxo-olean-12-ene-30-oic acid* (**13**)

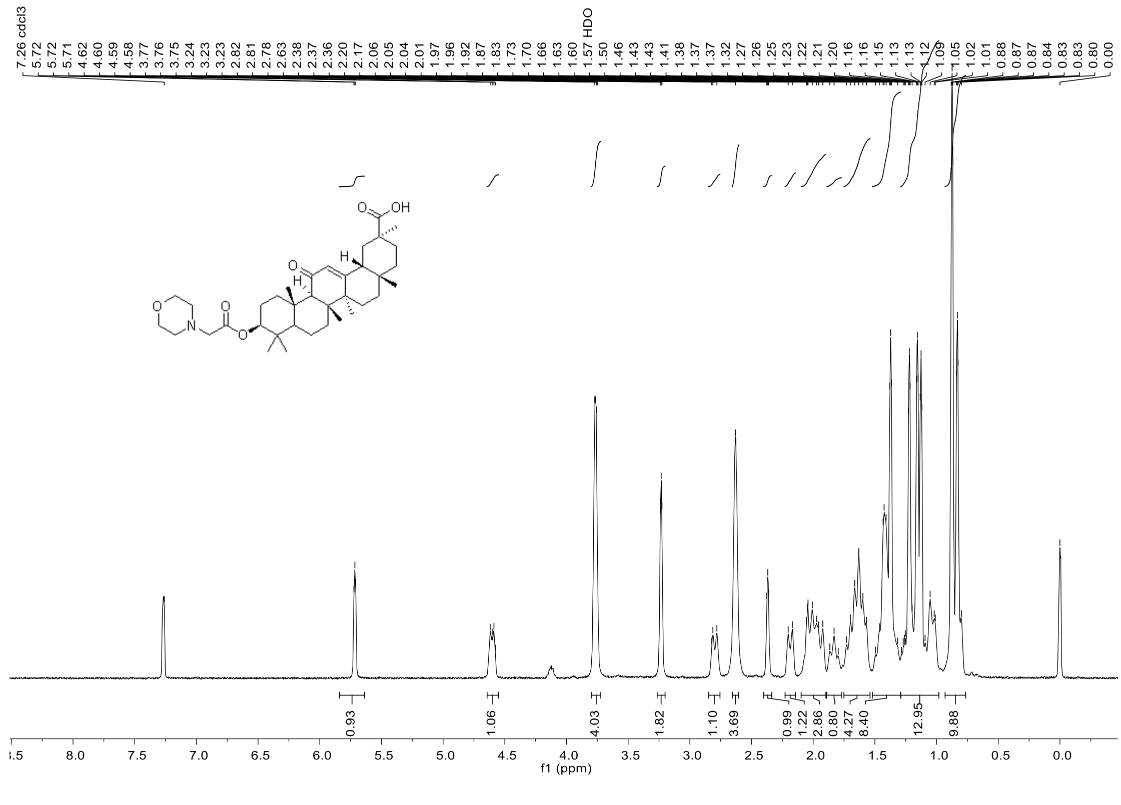

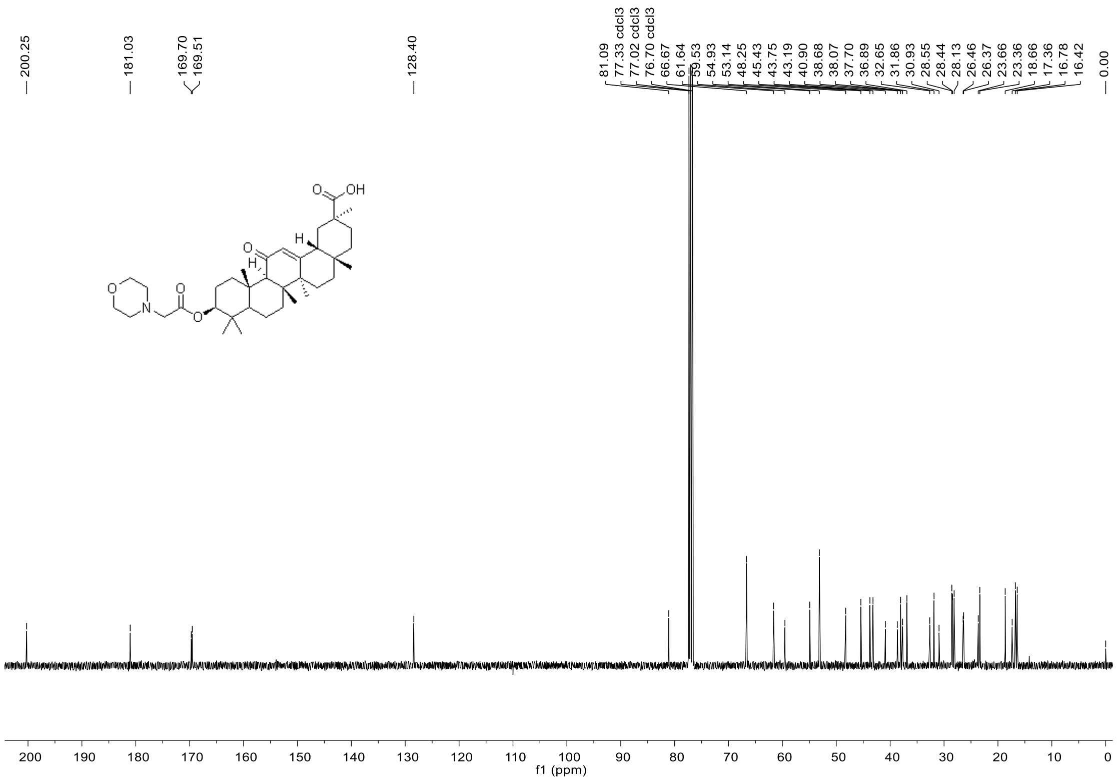
200.25
181.03
169.70
169.51
128.40
81.09
77.33 cdcl3
77.02 cdcl3
76.70 cdcl3
66.67
61.64
59.53
54.93
53.14
48.25
45.43
43.75
43.19
40.90
38.68
38.07
37.70
36.89
32.65
31.86
30.93
28.55
28.44
28.13
26.46
26.37
23.66
23.36
18.66
17.36
16.78
16.42
0.00
OH
O
H
O
H
O
N
O
O
200
190
180
170
160
150
140
130
120
110
100
90
80
70
60
50
40
30
20
10
0
f1 (ppm)

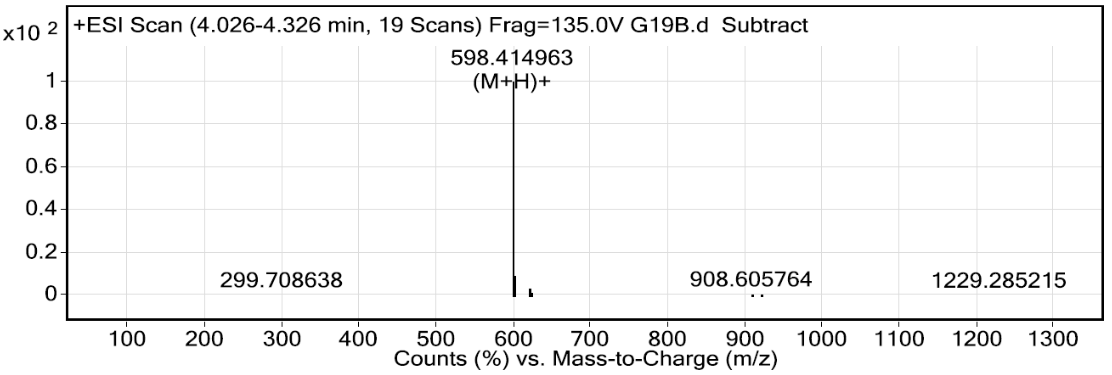
x10 2
+ESI Scan (4.026-4.326 min, 19 Scans) Frag=135.0V G19B.d Subtract
598.414963
(M+H)+
299.708638
908.605764
1229.285215
1
0.8
0.6
0.4
0.2
0
100
200
300
400
500
600
700
800
900
1000
1100
1200
1300
Counts (%) vs. Mass-to-Charge (m/z)

*4-(3β-(2-Morpholinoacetoxy)-11-oxo-18β-olean-12-en-30-carbonyl)piperazine-1-carboxylic acid* (**16**)

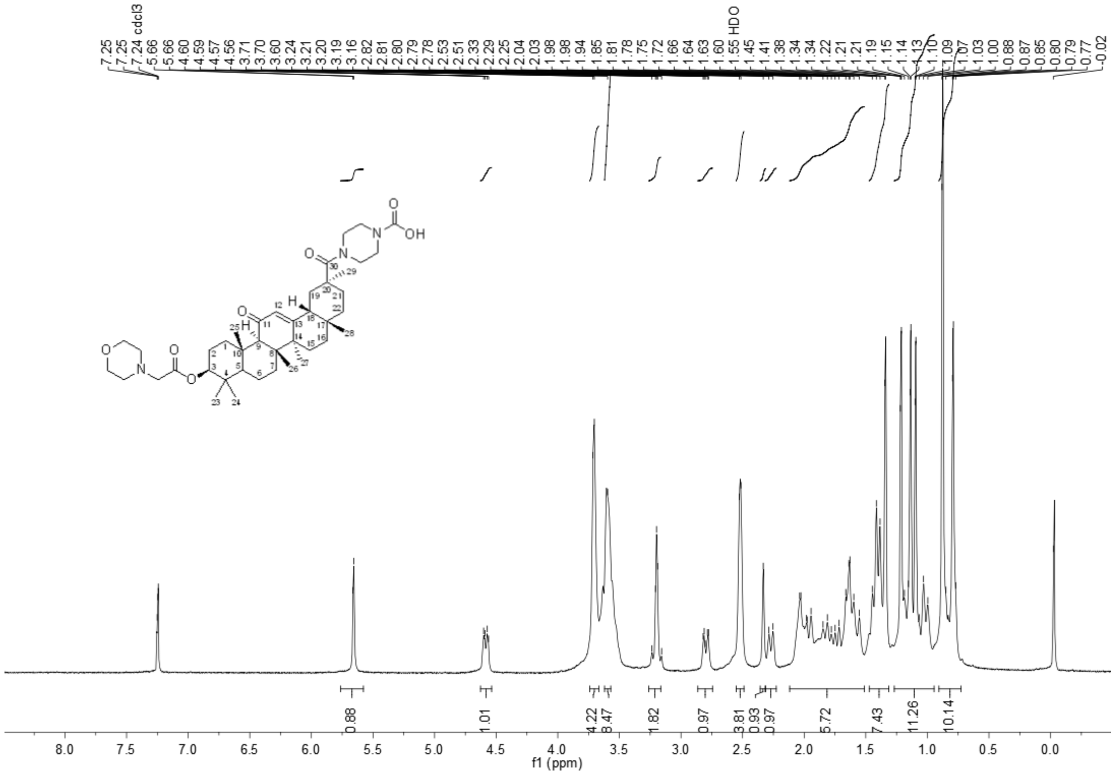

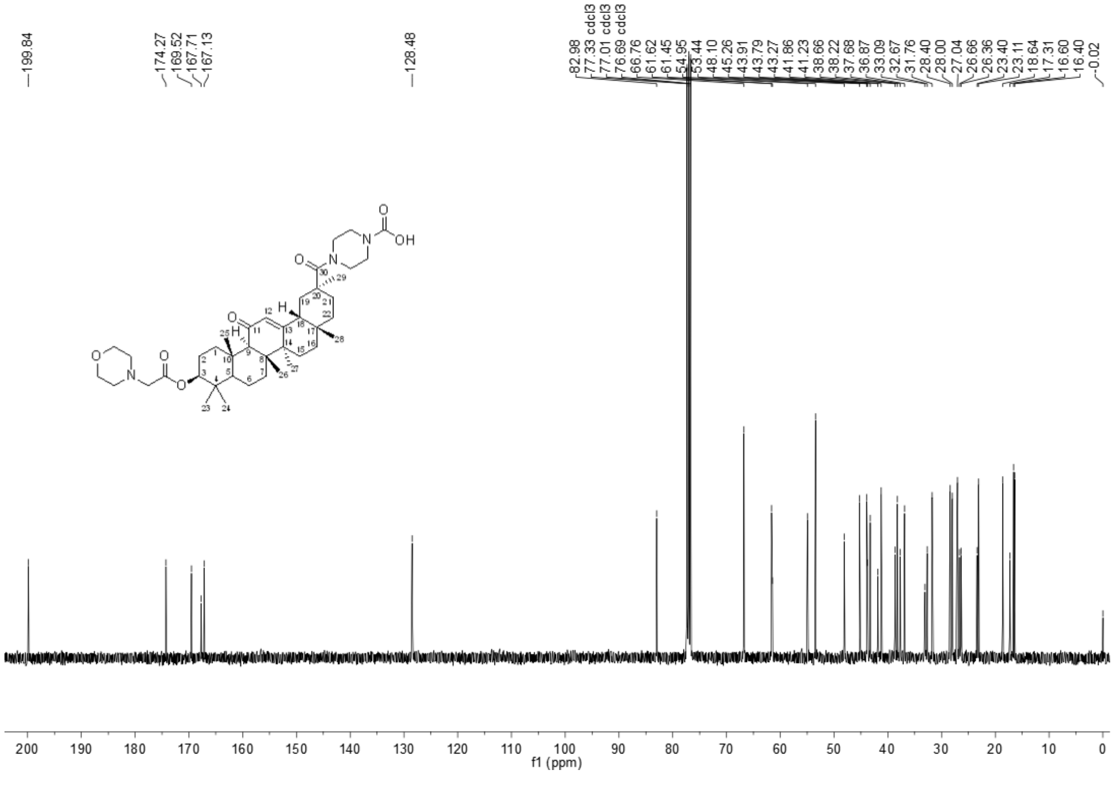

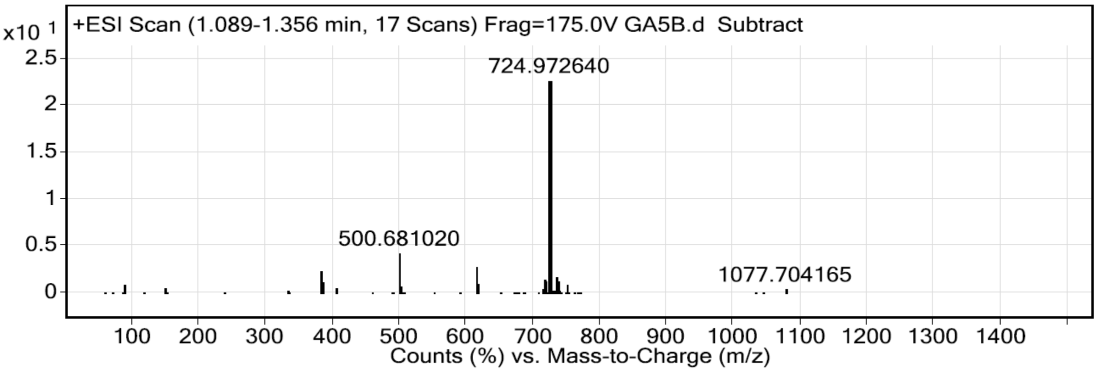


*3β-Acetoxy-11-oxo-18β-olean-12-en-30-carbonyl piperazine* (**4**)

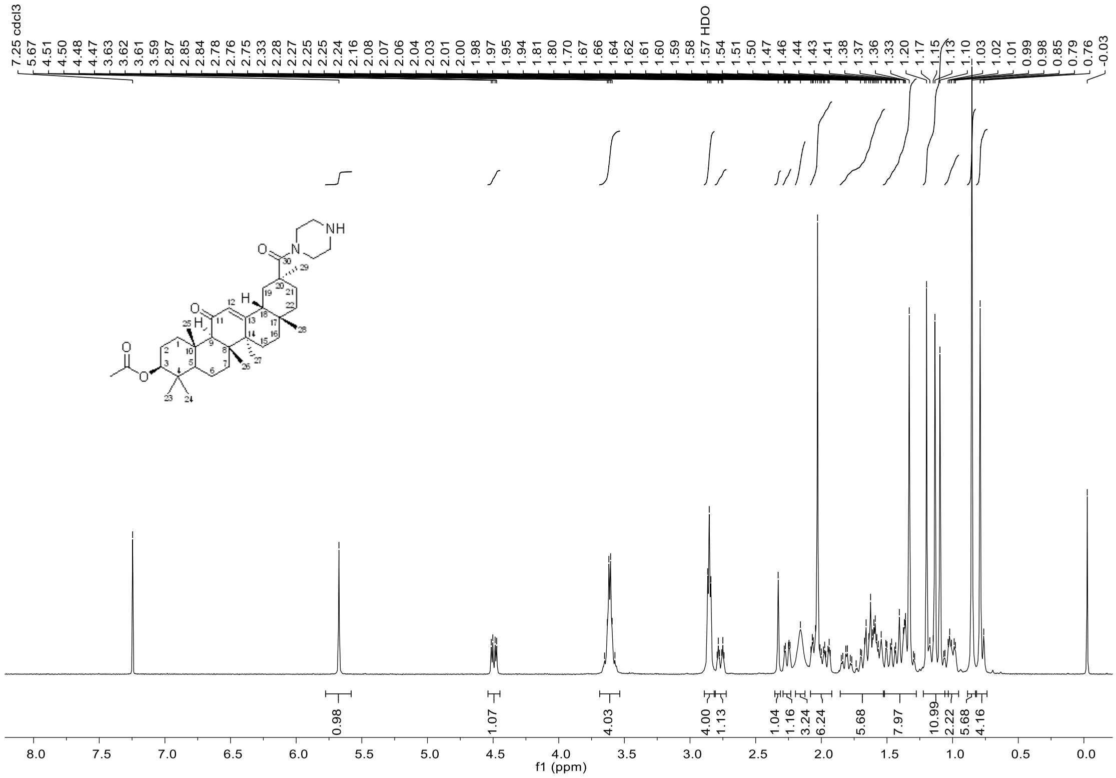

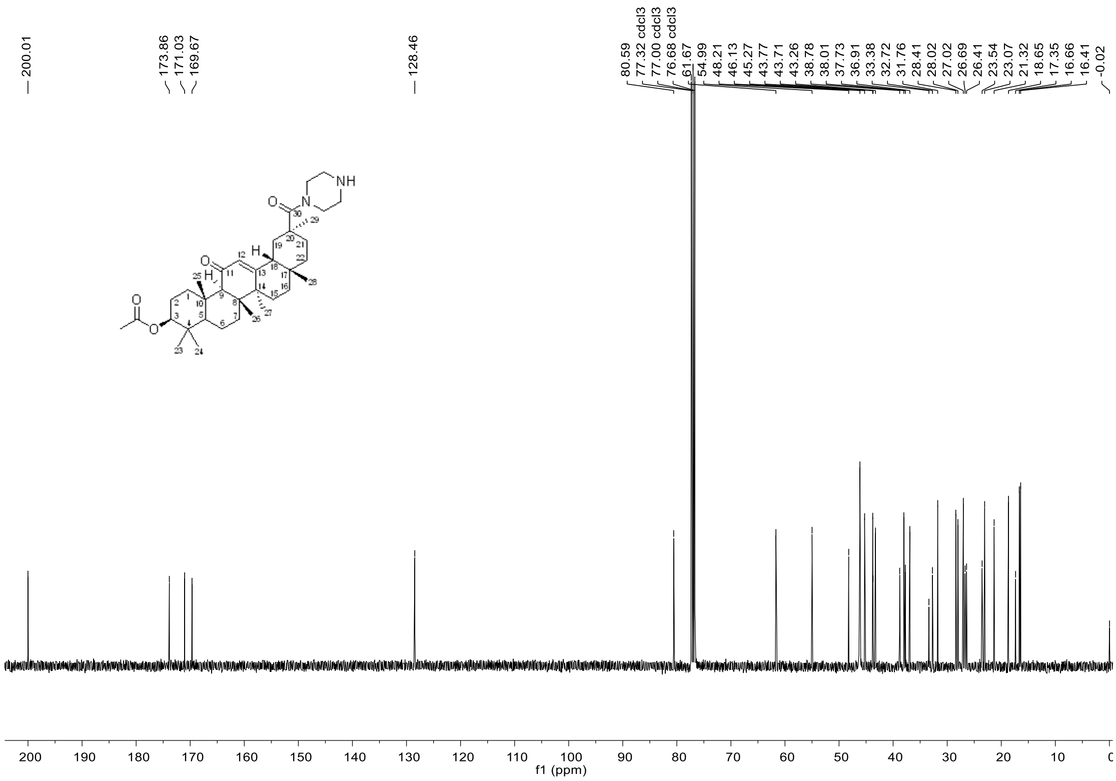


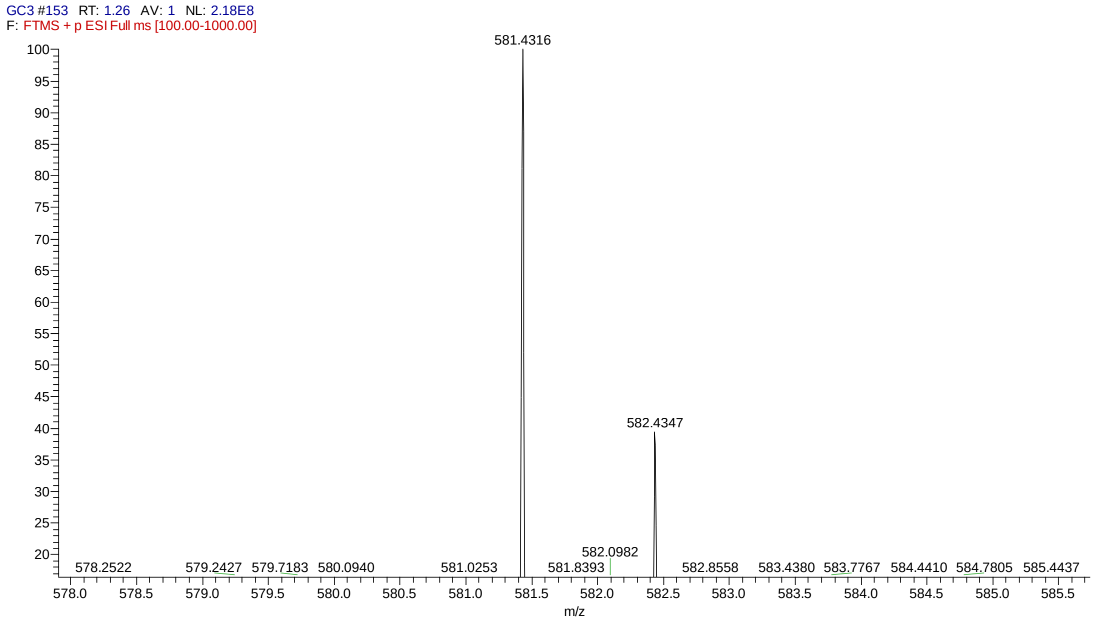


Bisamide product **5**

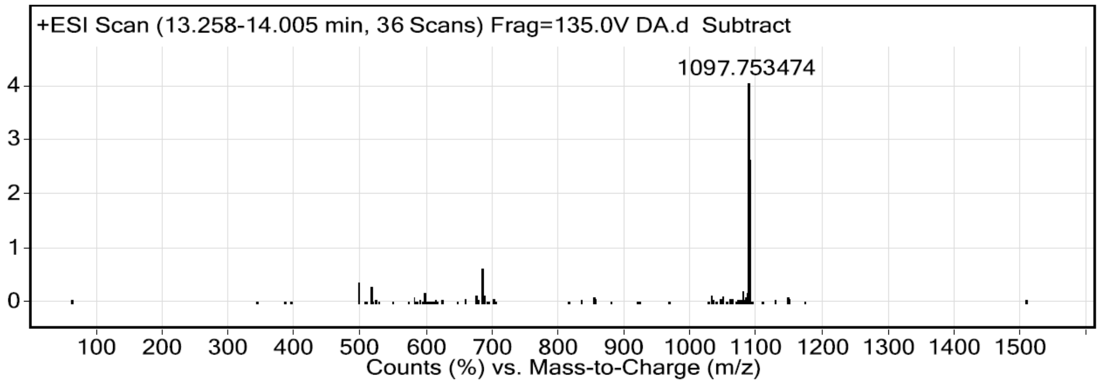

*3β-(2-Morpholinoacetoxy)-11-oxo-18β-olean-12-en-30-carbonyl piperazine* (**17**)

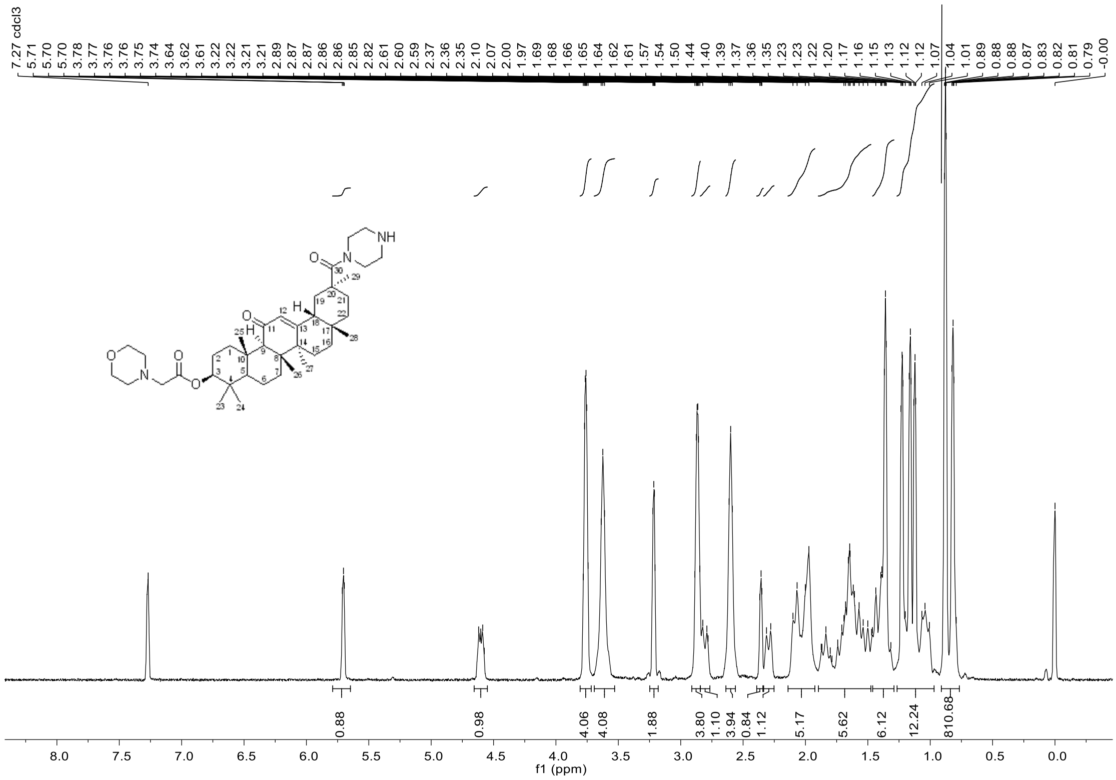

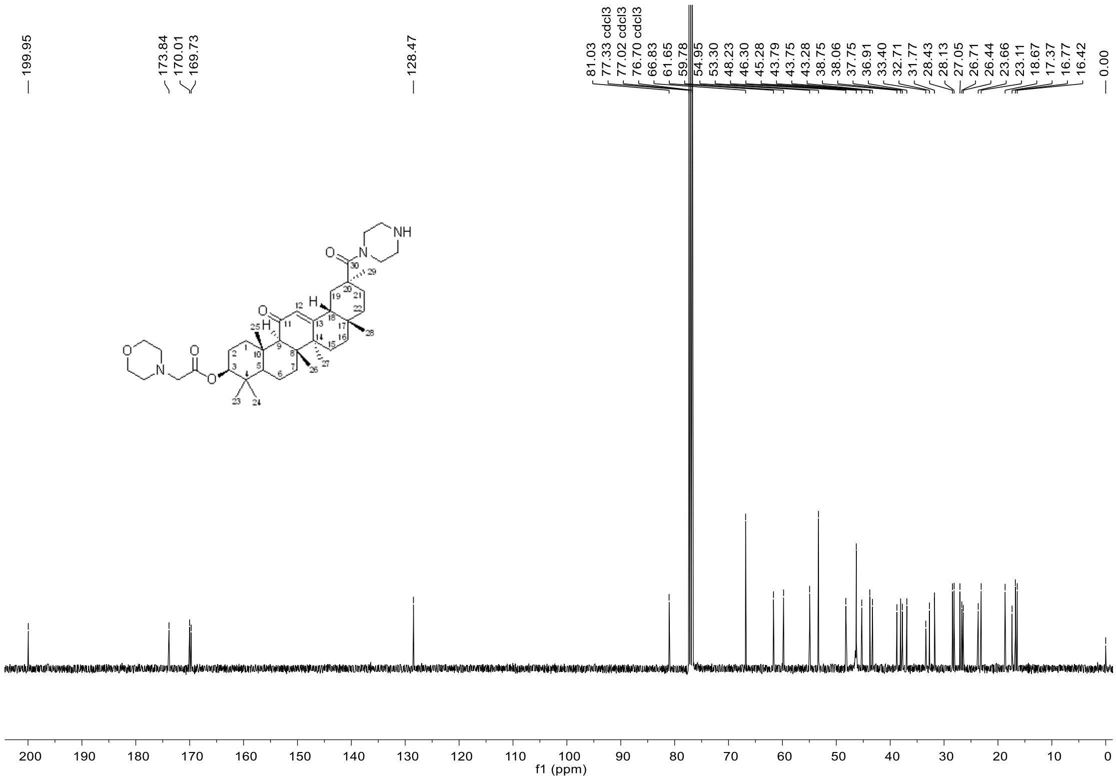

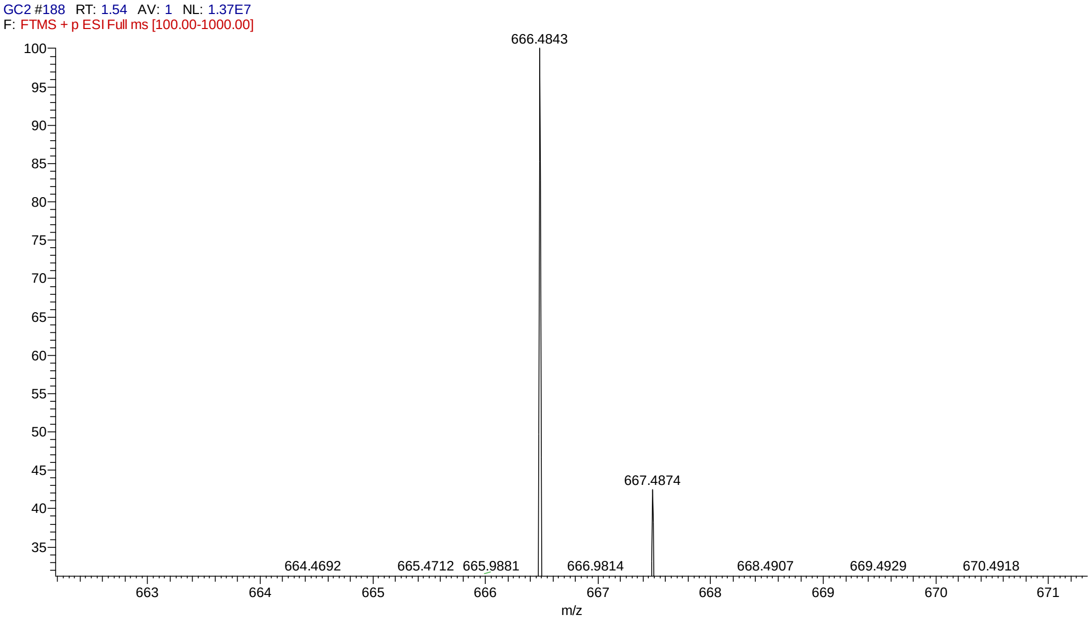


*3β-Acetoxy-30-(4-(3-fluorobenzoyl)-1-piperazinyl)-olean-12-ene-11,30-dione* (**18**)

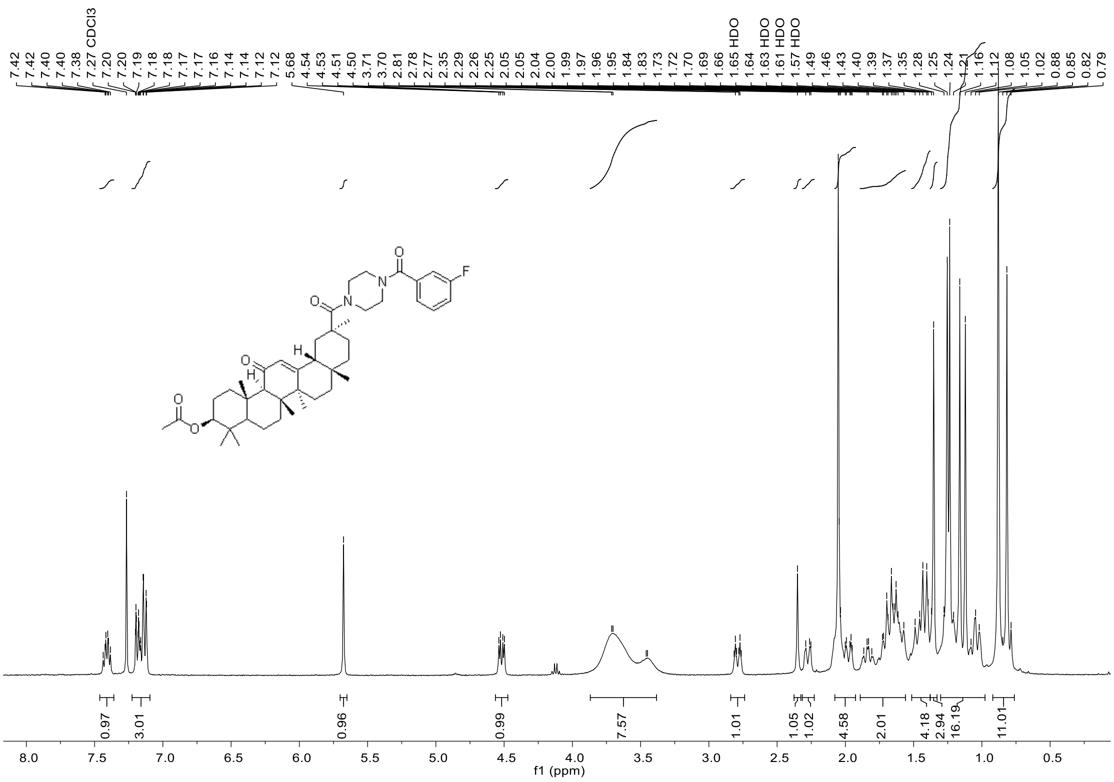

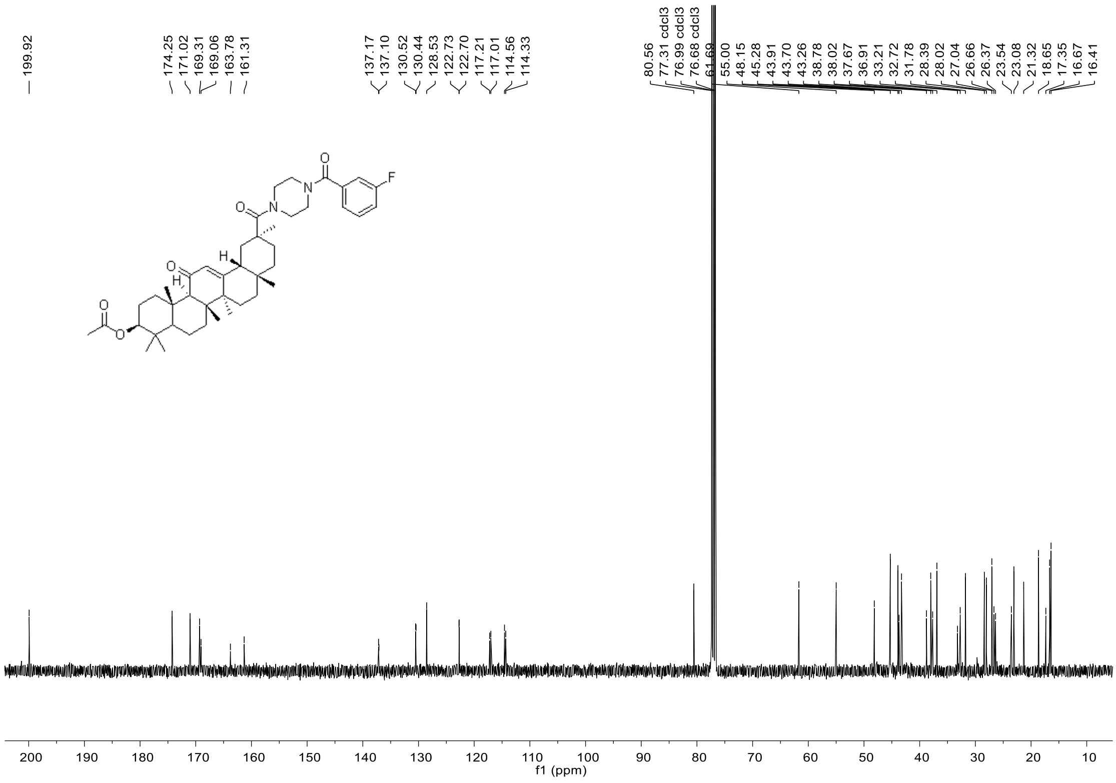

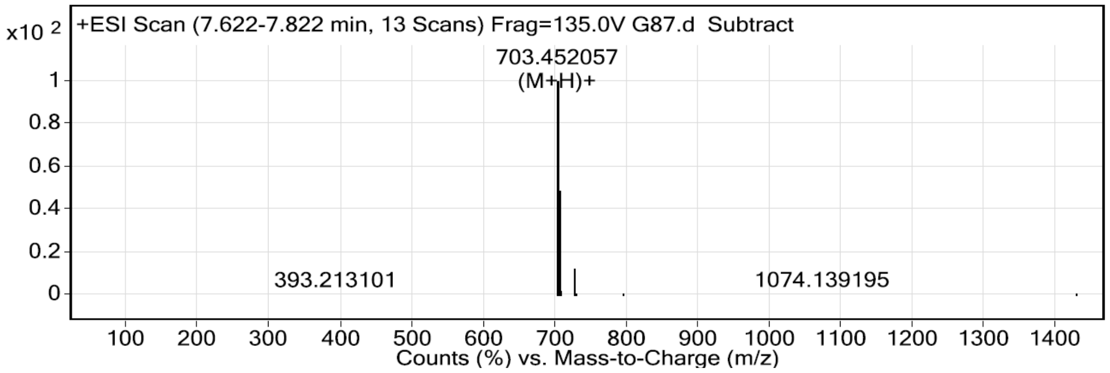


1. Song, H.; Sun, Y.; Xu, G.; Hou, B.; Ao, G. Synthesis and biological evaluation of novel hydrogen sulfide releasing glycyrrhetic acid derivatives. *J. Enzyme Inhib. Med. Chem.* **2016,** *31* (6), 1457-1463.

2. Sommerwerk, S.; Heller, L.; Kerzig, C.; Kramell, A. E.; Csuk, R. Rhodamine B conjugates of triterpenoic acids are cytotoxic mitocans even at nanomolar concentrations. *Eur. J. Med. Chem.* **2017,** *127*, 1-9.
